# PUS1 Drives Renal Cancer Progression by Preventing Formation of Endogenous Double-stranded RNAs

**DOI:** 10.7150/ijbs.130175

**Published:** 2026-03-25

**Authors:** Ziwei Zhu, Zeyi Lu, Fan Li, Zhehao Xu, Ruyue Wang, Yang Li, Haohua Lu, Yiming Ding, Wenqin Luo, Yudong Lin, Yi Lu, Xudong Mao, Mengxuan Li, Ziyuan Wang, Lifeng Ding, Liqun Xia, Gonghui Li

**Affiliations:** 1 Department of Urology, Sir Run Run Shaw Hospital, Zhejiang University School of Medicine, Hangzhou, 310016, China.; 2 Department of General Surgery, Sir Run Run Shaw Hospital, Zhejiang University School of Medicine, Hangzhou, 310016, China.

**Keywords:** renal cell carcinoma, PUS1, pseudouridine modification, translation, dsRNA, innate immune response

## Abstract

Pseudouridine (Ψ) modification is a prevalent epitranscriptomic mark with critical roles in carcinogenesis; however, the function of its catalytic "writer" enzyme, pseudouridine synthase 1 (PUS1), in renal cell carcinoma (RCC) remains elusive. Our analysis revealed that *PUS1* mRNA is upregulated in RCC and is associated with an unfavorable prognosis. Strikingly, this transcriptional upregulation results in a concomitant and exclusive increase in the protein abundance of PUS1 isoform 2. Mechanistically, although PUS1 markedly enhances global mRNA translation, this effect is not directly mediated via Ψ modification of either mRNA or tRNA. Instead, PUS1 regulates pre-mRNA splicing, and its deficiency induces elevated intron retention. This, in turn, culminates in the formation of double-stranded RNA (dsRNA), which subsequently activates the innate antiviral immune response and inhibits global translation. Furthermore, depletion of PUS1 in tumor cells significantly sensitizes RCC to immune checkpoint blockade therapy. Collectively, our findings demonstrate that PUS1 shields tumor cells from endogenous dsRNA accumulation and the consequent detrimental innate immune activation, thereby unveiling a novel and promising therapeutic strategy for RCC.

## 1. Introduction

Renal cell carcinoma (RCC) is a lethal malignant tumor arising from epithelial cells of the renal tubules, accounting for approximately 5% and 3% of all cancer cases in males and females, respectively^1^. RCC remains largely refractory to conventional chemotherapy and radiotherapy^2^, ^3^. Consequently, targeted therapy and immunotherapy constitute the mainstay of systemic treatment for RCC. Several tyrosine kinase inhibitors (TKIs) and immune checkpoint inhibitors (ICIs) have been approved for the management of advanced RCC^4^, ^5^. Although combination therapies integrating ICIs, represented by PD-1/PD-L1 inhibitors, with TKIs such as axitinib and sunitinib, have demonstrated efficacy in prolonging progression-free survival in advanced RCC, the five-year overall survival (OS) rate remains modest at approximately 40%^6^. The therapeutic development for these two drug classes may be approaching a plateau in RCC. Therefore, there is an urgent need for novel therapeutic strategies for advanced RCC, particularly those targeting pathways independent of PD-1/PD-L1 or angiogenesis.

Pseudouridine (Ψ), an abundant RNA modification formed through the isomerization of uridine, is ubiquitously present in tRNA and rRNA, and represents the second most prevalent modification in mRNA, following N6- methyladenosine (m6A)^7^, ^8^. Ψ is catalyzed by dyskerin and pseudouridine synthases (PUSs). By altering the C-C glycosidic bond conformation, Ψ enhances the hydrogen-bonding capacity of RNA, thereby modulating its stability, structure, and function^9^. Driven by the rising prominence of epitranscriptomics in oncology and the advent of pseudouridine-sequencing, the functions of pseudouridine synthase family in cancer progression are now coming to the forefront. For instance, dyskerin can synergistically suppress colorectal cancer cell growth with MEK1/2^10^. PUS7 is essential for the growth and survival of MYC-driven cancer by promoting adaptive stress responses and facilitating amino acid biosynthesis and import^11^. PUS10 regulates NUDC/Cofilin1 dependent renal cell carcinoma migration by promoting the maturation of miR-194-5p^12^. However, as the earliest discovered member of the PUSs, PUS1 can modify pre-mRNA affecting its processing^13^, introduce Ψ at mRNA stop codons to promote translational readthrough^14^, and catalyze consecutive Ψ27-28 sites modifications in human tRNA^15^, yet research in the field of oncology remains limited.

Double-stranded RNA (dsRNA) has long been considered to be associated exclusively with viral infections, including both RNA and DNA viruses. However, increasing evidence indicates that under various pathophysiological conditions, endogenous sources, such as retrotransposable elements and mitochondrial DNA, can also generate dsRNA^16^. Transcriptional derepression, achieved through the suppression of DNA methylation, can lead to dsRNA formation^17^. Loss of key mitochondrial RNA degradosome components, such as PNPase or SUV3, causes mitochondrial dsRNA accumulation^18^. Adenosine-to-inosine (A-to-I) editing destabilizes dsRNA structures, and the lack of ADAR1 leads to a reduction in the disruption of these dsRNAs^19^. These endogenous dsRNA activate the same receptors as viral dsRNA, thereby triggering innate immune responses within tumor cells. This mechanism represents a promising avenue for enhancing the efficacy of cancer immunotherapy.

In this study, we found that *PUS1* mRNA is upregulated in RCC and associated with poor prognosis. Strikingly, this transcriptional upregulation leads to a concomitant increase exclusively in the protein levels of PUS1 isoform 2. Furthermore, upregulated PUS1 isoform 2 enhances RCC cell proliferation both *in vitro* and *in vivo*, an activity that is dependent on its pseudouridylation catalytic function. Mechanistically, although PUS1 markedly promotes global mRNA translation, this effect is not directly mediated by Ψ modification of either mRNA or tRNA. We discovered that PUS1 is a key regulator of endogenous dsRNA levels. Knockdown of PUS1 leads to increased intron retention, producing more dsRNA, which in turn inhibits global translation and activates the innate immune system. Based on these findings, we combined a PUS1-targeting strategy with ICIs. This combination therapy synergistically enhanced the infiltration of CD4^+^ and CD8^+^ T cells and suppressed RCC progression. In summary, our research provides a novel and promising treatment option for RCC.

## 2. Materials and Methods

### 2.1 Clinical Specimens

Renal cell carcinoma (RCC) specimens for this study cohort were obtained from Sir Run Run Shaw Hospital, Zhejiang University School of Medicine, with approval from its Ethics Committee (SRRSH20230211). Informed consent was obtained from all patients. Detailed clinical characteristics of the RCC patients are listed in Supplementary Table S2.

### 2.2 Cell Culture and Transfection

Renal cell carcinoma cell lines (786-O, ACHN and Renca) were purchased from the Cell Bank of Type Culture Collection of the Chinese Academy of Sciences. The 786-O and Renca cell line was cultured in RPMI-1640 medium supplemented with 10% fetal bovine serum (FBS, Cellmax, China). The ACHN cells were cultured in MEM medium supplemented with 10% FBS. All cells were maintained at 37°C incubator with 5% CO₂.

Plasmid and siRNA transfections were performed using jetPRIME (Polyplus, 101000046) according to the manufacturer's instructions. siRNAs were synthesized by Genepharma (China); plasmids were constructed and produced by CORUES (China). For stable transfection, lentiviruses were designed, synthesized, and collected by Genepharma (China), and used to infected RCC cells with the provided transfection reagent. Stable infected cell lines were subsequently selected using puromycin (Selleck, E4996) or Blasticidin S (Yeasen, 60218ES10). All sequences are listed in Supplementary Table S3.

### 2.3 Animal experiment

The animal experiment procedures were approved by the Ethics Committee of Sir Run Run Shaw Hospital, Zhejiang University School of Medicine (SRRSH202302221).

For the subcutaneous implantation model, 2 × 10⁶ 786-O cells were subcutaneously injected into 4-week-old BALB/c nude mice. Tumor volume was measured every 3 days using the formula: Volume = (Length × Width²) / 2. Tumors were collected and weighed at specified time points. Alternatively, 1 × 10⁶ Renca cells were subcutaneously injected into 6-week-old male BALB/c mice. Tumor volume was measured daily, and tumors were collected, weighed at specific time points, and their immunophenotypes assessed by flow cytometry. To evaluate the efficacy of immune checkpoint antibodies, intraperitoneal injections of 15 µg/g control IgG2a (Selleck, A2123) or anti-CD279 (Selleck, A2122) antibody were administered (starting on designated days, every 3 days).

For the renal orthotopic implantation model, 2 × 10⁶ ACHN cells were injected beneath the renal capsule of 4-week-old BALB/c nude mice. After an appropriate period, mice were anesthetized, and tumor growth and metastasis were detected using an *in vivo* imaging system (IVIS).

### 2.4 RNA extraction and quantitative real-time PCR (qRT-PCR)

Total RNA was extracted from RCC tissues or cells using TRIzol reagent (CWbio, CW0580S) according to the manufacturer's instructions. cDNA was synthesized after genomic DNA removal using the HiFiScript gDNA Removal RT MasterMix (CWbio, CW2020M). qRT-PCR analysis was performed using SYBR Green (CWbio, CW0957). All primers were synthesized by TsingKe Biotechnology (China). Detailed primer information is provided in Supplementary Table S4.

### 2.5 Cell counting kit-8 (CCK-8) assay

Transfected RCC cells were seeded into 96-well plates at a density of 1,000 cells per well. At 1, 2, 3, 4, and 5 days post-seeding, CCK-8 reagent (Glpbio Technology, GK10001) was added to each well. After incubation at 37°C with 5% CO₂ for 2 hours, the absorbance at 450 nm was measured using a Multiskan FC microplate photometer (Thermo Scientific).

### 2.6 Colony formation assay

Transfected RCC cells were seeded into 6-well plates at a density of 1,000 cells per well. After an appropriate duration, cells were fixed with 4% paraformaldehyde, stained with 0.3% crystal violet, and imaged using a SYSTEM GelDoc XR (Bio-Rad).

### 2.7 EdU incorporation assay

The EdU incorporation assay was performed using the EdU Cell Proliferation Kit (with Alexa Fluor 555, Meilunbio, MA0425-1). Transfected RCC cells were seeded into 96-well plates at 5,000 cells per well. After cell attachment, the medium was supplemented with 10 µM EdU and incubated overnight. Cells were then fixed with 4% paraformaldehyde, permeabilized with 0.1% Triton X-100, and incubated with a small azide molecule labeled with fluorescence dyes, followed by Hoechst staining. Images were acquired using a fluorescence microscope (Olympus).

### 2.8 Flow cytometry

For cell cycle analysis, transfected RCC cells were collected and washed twice. Cells were then stained with Propidium Iodide staining solution (Multi Sciences, CCS012) and analyzed after 30 minutes of incubation using a flow cytometer (Beckman, CytoFLEX LX).

For tumor-infiltrating lymphocyte analysis, tumor tissues were collected, minced, and digested with Collagenase IV and DNase I at 37 °C for 15 minutes. Single-cell suspensions were obtained by filtering through a 70 µm strainer, followed by red blood cell lysis and Percoll (Cytiva, 17089109) gradient centrifugation. Cells were stained with antibody cocktails against CD45, CD3, CD4, and CD8a (details in Supplementary Table S5) and analyzed using a flow cytometer to determine immune cell proportions.

### 2.9 Wound healing assay

Transfected RCC cells were seeded into 6-well plates. Upon reaching 90% confluence, a linear wound was created using a 200 µL pipette tip. Wound images were captured at 0 and 24 hours using a microscope.

### 2.10 Transwell assay

A total of 1 × 10⁴ transfected RCC cells resuspended in 200 µL of FBS-free medium were seeded into the upper chamber of an 8 µm pore size Transwell insert (Corning, USA). The lower chamber was filled with 200 µL of medium containing 10% FBS. After 24 hours, migrated cells were fixed and stained with 0.3% crystal violet.

### 2.11 Western blotting

Cells or tissues were lysed using RIPA lysis buffer, and proteins were denatured at 98°C for 20 minutes. Proteins were separated by SDS-PAGE and transferred onto a 0.2 µm PVDF membrane (Bio-Rad, 1620177). After blocking with 5% skim milk, the membrane was incubated with primary antibodies at 4 °C overnight, followed by incubation with corresponding secondary antibodies. Signals were detected using an ECL kit (FDbio, FD8000/FD8030). Details of antibodies are listed in Supplementary Table S5.

### 2.12 Dot blot assay

RNA was denatured at 95 °C for 3 minutes to remove secondary structures and then blotted onto an N+ membrane (GE Healthcare, RPN303B). After UV cross-linking, the membrane was washed with PBS containing 0.02% Tween, blocked with 5% skim milk, and incubated with an anti-pseudouridine antibody at 4 °C overnight. The membrane was then incubated with a secondary antibody, and signals were detected using an ECL system. Total RNA loading was assessed using 0.02% methylene blue staining.

### 2.13 RNA immunoprecipitation (RIP)

RIP was performed using the Magna RIP Kit (Millipore, MAGNARIP01) according to the manufacturer's instructions. Briefly, 2 × 10⁷ RCC cells were lysed in RIP lysis buffer. The lysate was incubated with primary antibody or IgG and magnetic Protein A/G beads at 4 °C overnight. The antibody-RNA complexes were washed five times, digested with Proteinase K, and the immunoprecipitated RNA was purified.

### 2.14 Immunohistochemistry (IHC) staining

Sections were deparaffinized, rehydrated, and subjected to microwave-assisted antigen retrieval. After blocking endogenous peroxidase and nonspecific sites, slides were incubated with primary antibody at 4 °C overnight, followed by HRP-conjugated secondary antibody. DAB was used for chromogenic development, with hematoxylin counterstaining. Finally, sections were dehydrated, cleared, and mounted for microscopic examination.

IHC score was performed independently by two senior pathologists, with the pathology department director serving as the final arbitrator for any discrepancies.

### 2.15 Immunofluorescence

RCC cells seeded on glass coverslips in 24-well plates were fixed with 4% paraformaldehyde, permeabilized with 0.5% Triton X-100, blocked with 5% BSA, and incubated with primary antibody at 4 °C overnight. After washing, cells were incubated with fluorescent secondary antibody at room temperature for 1 hour, followed by DAPI staining. Images were acquired using a confocal microscope (Olympus FV3000). For J2 intensity analysis, signals were quantified using ImageJ and normalized to DAPI signals. For mitochondrial staining, cells were incubated with 100 nM Mito-Tracker Red CMXRos (Beyotime, C1049B) at 37 °C for 20 minutes before fixation.

### 2.16 Polysome profiling

A total of 2 × 10⁷ RCC cells were treated with 100 µg/mL cycloheximide (MCE, 66-81-9) at 37°C for 5 minutes, washed with PBS containing 100 µg/mL cycloheximide, and lysed on ice for 5 mins with 400 µL of polysome lysis buffer (1% Triton X-100, 0.3 m NaCl, 15 mm MgCl2, 15 mm Tris-HCl (pH 7.4), 100 µg mL-1 cycloheximide, 1 mm DTT and 100U RNase inhibitor(Takara, 2313B)). Centrifuged at 12,000 rpm at 4 °C for 10 min, the supernatant was added to the sucrose density gradient and centrifuged at 4 °C at 38,000 rpm for 120 min (Beckman , Optima XPN-100). Gradients were fractionated using a gradient fractionation system (Biocomp), and absorbance at 260 nm was recorded.

### 2.17 Mitochondrial isolation

Mitochondria were isolated using the Mitochondrial Isolation Kit (Beyotime, C3601) according to the manufacturer's instructions. Briefly, harvest cells and wash with ice-cold PBS. Resuspend the cell pellet in Mitochondria Isolation Reagent and incubate on ice for 10-15 minutes. Homogenize the suspension with a glass homogenizer (10-30 strokes). The homogenates were centrifuged at 600 g at 4 °C for 10 min, and the supernatants were collected and centrifuged another 600 g for 10 min for more purity. Last, the supernatants were centrifuged at 11000 g for 10 min and the pellet obtained was the isolated mitochondria.

### 2.18 Nascent protein synthesis assay

Nascent protein synthesis was assessed using the BeyoClick™ HPG Protein Synthesis Kit with Alexa Fluor 488 (Beyotime, P1202S) according to the manufacturer's instructions. Cells were incubated with 1x HPG solution for 30 minutes. Fixed with 4% PFA for 15 min, permeabilized with 0.3% Triton X-100, then incubated with a fluorescent azide probe, followed by Hoechst staining. Images were acquired using a fluorescence microscope (Olympus). HPG signal intensity was quantified using ImageJ and normalized to Hoechst signal.

### 2.19 Ribo-seq analysis and codon occupancy analysis

2 × 10⁷ RCC cells were collected from each sample for ribosome profiling. Cells were washed twice with ice-cold PBS containing 100 µg/mL cycloheximide, and cell pellets were snap frozen with liquid nitrogen. Ribosome footprint extraction and library construction were conducted at Novogene. Briefly, ribosome-protected footprints were isolated from cell lysates treated with cycloheximide. Following RNase I digestion, monosomes were purified by size-exclusion chromatography. Ribosome-protected footprints (20-38 nt) were size-selected by PAGE, ligated with adapters, and converted to sequencing libraries. Libraries were sequenced on Illumina platforms (PE150). After quality control including adapter trimming and quality filtering, clean reads were aligned to the reference genome using TopHat2. Gene expression quantification was performed with HTSeq, and differential expression analysis was conducted using DESeq2 (for samples with replicates), with |log2FC| > 0.5 and p-value < 0.05 set as significance thresholds.

To examine the codon occupancy on the ribosome A site, clean reads were aligned to the H. sapiens genome (GRCh38), following CONCUR pipeline analysis^20^ and codon enrichment calculation and normalization as described^21^. The differences of the codon occupancy on the ribosome A site between different conditions were calculated as the differences of normalized codon counts.

### 2.20 Quantitative proteomics

4 × 10^6^ RCC cells were collected from each sample for quantitative proteomics. Cells were lysed using 8M urea buffer, and protein concentration was determined by BCA assay. Proteins (100 μg) were reduced with DTT, alkylated with iodoacetamide, and digested with trypsin overnight. Peptides were desalted using C18 columns and analyzed by nano-UHPLC coupled to an Orbitrap Astral mass spectrometer operating in DIA mode. MS data were processed using DIA-NN (v1.8.1) with library-free search against the human UniProt database. Protein quantification was performed using the MaxLFQ algorithm, with intensity normalization to median values. Proteins were considered differentially expressed with FC > 1.3 or <1/1.3 and p-value < 0.05.

### 2.21 Codon usage analysis

Homo sapiens GRCh38 CDS sequences were obtained from the Ensembl database (Ensembl Genes 105). Principal splice isoforms were filtered using annotations from the APPRIS database (Genecode 39, Ensembl 105)^22^, selecting the highest-scoring transcript for genes with multiple isoforms. FASTA sequences were processed using the SeqinR package, and codon usage frequency was calculated with CoRdon (v1.16.0) in R.

### 2.22 RNA sequencing and intron retention analysis

Total RNA was extracted and assessed for quality. Strand-specific sequencing libraries were prepared through mRNA enrichment, fragmentation, and cDNA synthesis. Libraries were sequenced on the Illumina NovaSeq X Plus (Novogene). Clean reads were aligned to the reference genome using HISAT2, and gene expression levels were quantified. Differential expression analysis was performed using DESeq2, with |log2(fold change)| ≥ 1 and adjusted p-value < 0.05 considered statistically significant.

Intron retention (IR) scores were calculated using the sam2irs pipeline (https://github.com/rwanwork/sam2irs). Briefly, aligned reads were first filtered to retain valid exons by excluding unwanted chromosomes, "Gm"-prefixed genes, and non-exon features. Subsequently, the pipeline processed each chromosome to define gene, intron, and exon regions, during which overlapping genes were discarded and isoform exons were collapsed. Finally, base alignments were counted utilizing the CIGAR strings of the aligned reads.

### 2.23 Statistical analysis

Data are presented as mean ± SD. The two-tailed Student's t test was performed to evaluate the differences between two groups, and one-way ANOVA was used for comparisons involving more than two groups in GraphPad Prism 10.6. Statistical significance was defined as * p < 0.05, ** p < 0.01, *** p < 0.001, and **** p < 0.0001.

## 3. Results

### 3.1 *PUS1* is upregulated in renal cell carcinoma and associated with poor clinical prognosis

**Figure 1:**
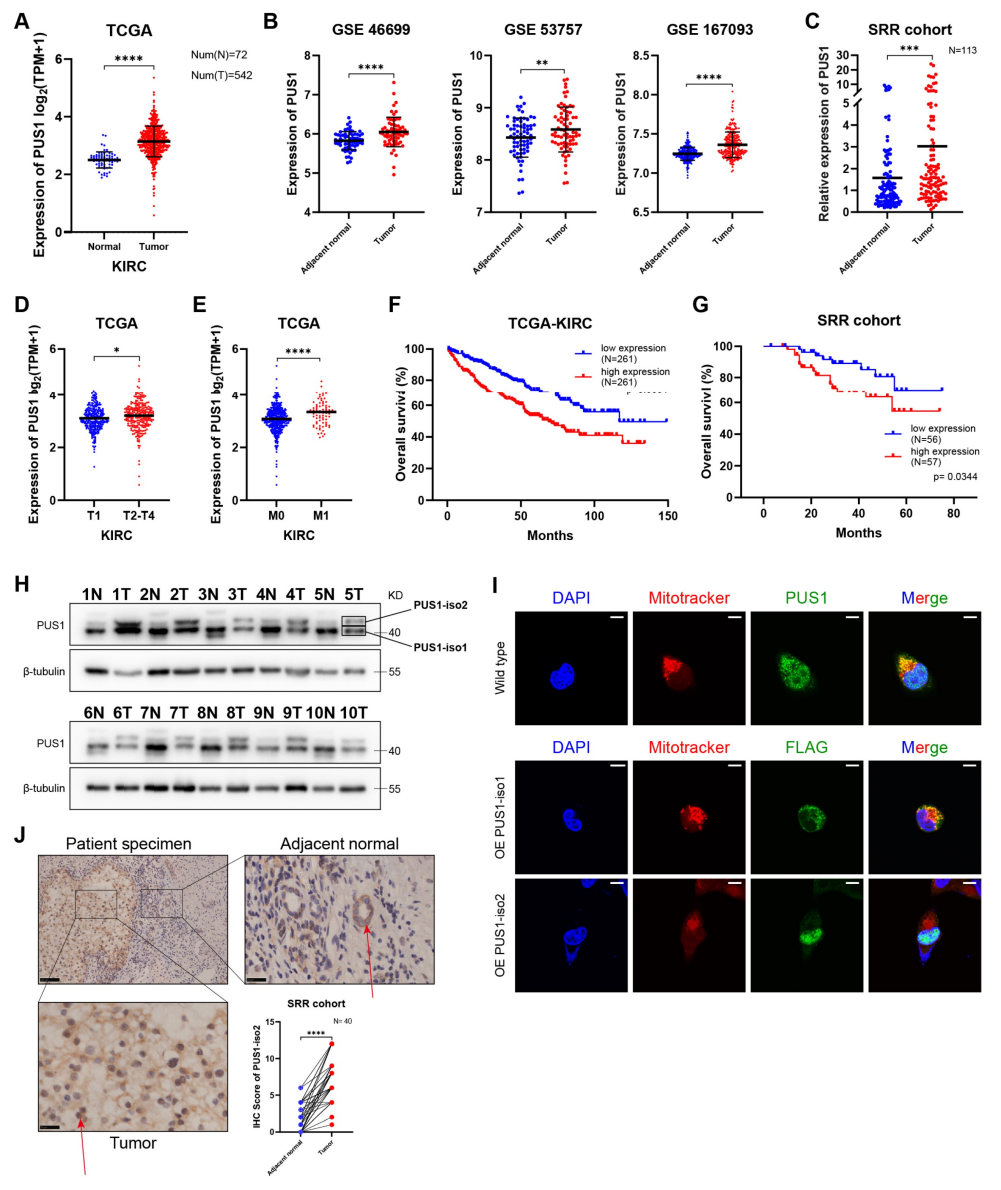
***PUS1* mRNA expression is significantly upregulated in RCC and associated with poor prognosis, whereas at the protein level, only PUS1 isoform 2 shows elevated expression.** (A) The mRNA expression of *PUS1* in KIRC was analyzed with the TCGA database. (B) *PUS1* mRNA expression in adjacent normal tissues and RCC specimens in GSE46699, GSE53757 and GSE167093. (C) RT-qPCR analysis of *PUS1* in RCC specimens and adjacent normal tissues from the Sir Run Run Shaw Hospital (SRR) RCC cohort. (D,E) *PUS1* expression level between T1 stage and T2-T4 stage (D), non-metastasis and metastasis groups (E) from the TCGA-KIRC cohort. (F,G) Kaplan‒Meier survival curves of overall survival in low and high* PUS1* expression groups from the TCGA-KIRC cohort (F) and the SRR RCC cohort (G). (H) Western blot analysis of the PUS1 protein expression in paired RCC tumor and normal tissues from the SRR RCC cohort; PUS1-iso1: PUS1 isoform 1; PUS1-iso2: PUS1 isoform 2. (I) Representative immunofluorescence images in 293T cells stained with anti-PUS1 or anti-FLAG. Nuclei were stained with DAPI. Mitochondria were stained by mitotracker. Scale bar, 10 µm. (J) Representative IHC staining images for PUS1 protein in the SRR RCC cohort are presented. IHC scores are calculated and analyzed. Arrows indicate the cell nuclei. *P < 0.05, **P < 0.01, ***P < 0.001, ****P < 0.0001; ns, not significant.

To investigate the role of pseudouridine (Ψ) writer PUS1 in renal cell carcinoma (RCC), we first analyzed its expression using public databases. In the TCGA-KIRC cohort, *PUS1* mRNA expression was significantly higher in tumor tissues compared to normal tissues (Figure 1A). Three GEO-RCC microarray datasets showed similar results (Figure 1B). This finding was also validated in our internal RCC cohort of 113 patients by RT-qPCR (Figure 1C).

Subsequently, we explored the correlation between *PUS1* expression and clinical parameters. PUS1 expression was significantly higher in patients with T2-T4 stage tumors compared to those with T1 stage (Figure 1D; Figure S1A). Additionally, *PUS1* expression was higher in metastatic RCC patients (Figure 1E; Figure S1B). For survival analysis, patients were dichotomized into high- and low-PUS1 expression groups based on the median expression value. The results showed that patients with high *PUS1* expression had a poorer prognosis in both TCGA-KIRC cohort and SRR cohort (Figure 1F, G). These data suggested that *PUS1* is upregulated in RCC tissues and correlates positively with malignant features.

However, a paradoxical finding emerged at the protein level. Western blot analysis from our internal RCC cohort revealed two distinct bands: an upper band consistently overexpressed in tumor tissues and a lower band with variable expression compared to adjacent normal tissues (Figure 1H). Literature review revealed that PUS1 has two isoforms: isoform 1 (located in mitochondria, corresponding to the lower band) and isoform 2 (primarily located in the nucleus, corresponding to the upper band)^23^. Mitochondrial isolation in 293T cells confirmed the specific subcellular localization, with only the isoform 1 band being detected in the mitochondrial fraction (Figure S1C). Moreover, immunofluorescence also validated the distribution of PUS1 (Figure 1I). Subsequent immunohistochemistry (IHC) on our RCC specimens confirmed the specific nuclear overexpression of PUS1 isoform 2 (Figure 1J; Figure S1D). IHC analysis revealed intense nuclear staining in tumor cells, in contrast to the predominant cytoplasmic staining observed in adjacent normal tissues. To elucidate this discrepancy, we designed transcript-specific primers to distinguish *PUS1* isoform 1 and isoform 2. Our results showed that both isoforms were significantly upregulated at the mRNA levels in patients (Figure S1E), indicating the presence of specific post-transcriptional dysregulation. Given the hypoxia in RCC tissues and the common presence of VHL mutations, leading to mitochondrial depletion^24^, we hypothesize that mitochondrial loss in RCC may contribute to the decreased protein levels of PUS1 isoform 1.

### 3.2 PUS1 is crucial for RCC progression

**Figure 2:**
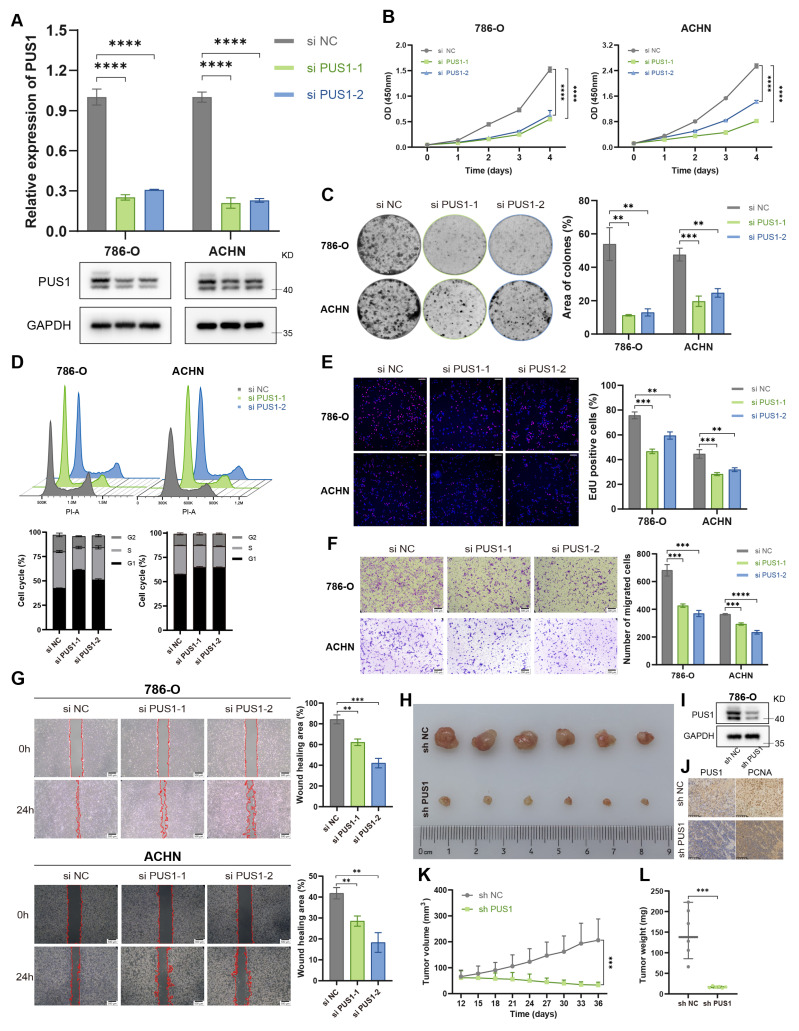
** PUS1 knockdown inhibits RCC progression *in vitro* and *in vivo*.** (A) RT-qPCR and western blot analysis confirming the knockdown of PUS1 in 786-O and ACHN cells. (B) CCK-8 assays were performed to determine cell proliferation upon PUS1 knockdown. (C) Representative images of colony-formation assay and its quantification data in 786-O and ACHN cells following PUS1 knockdown. (D) Flow cytometric analysis of cell cycle and its quantification data in 786-O and ACHN cells following PUS1 knockdown. (E) Representative images of EdU incorporation assay and its quantification data in 786-O and ACHN cells following PUS1 knockdown. Scale bar, 200µm. (F) Representative images of transwell migration assay and its quantification data in 786-O and ACHN cells following PUS1 knockdown. Scale bar, 200µm. (G) Representative images of wound healing assay in 786-O and ACHN cells following PUS1 knockdown. Scale bar, 200µm. (H) Images of xenograft tumors derived from the indicated cells in control and PUS1 knockdown groups (n = 6). (I) Western blot analysis showing the PUS1 depletion in 786-O cells transduced with lentivirus. (J) Representative IHC staining images for PUS1, PCNA of 786-O cell-derived xenograft tumors. Scale bar, 100 µm. (K) volumes of xenograft tumors derived from the indicated cells in control and PUS1 knockdown groups. (L) weights of xenograft tumors derived from the indicated cells in control and PUS1 knockdown groups. *P < 0.05, **P < 0.01, ***P < 0.001, ****P < 0.0001; ns, not significant.

To elucidate the role of PUS1 in the progression of RCC, we knocked down PUS1 expression in 786-O and ACHN using two independent siRNAs and confirmed the knockdown efficiency at both the mRNA and protein level (Figure 2A). Cell Counting Kit-8 (CCK-8) assays revealed that RCC cell proliferation was significantly impaired following PUS1 knockdown (Figure 2B). In addition, PUS1 knockdown markedly reduced colony-forming ability (Figure 2C). Cell cycle analysis indicated that PUS1 silencing induced G1 phase arrest (Figure 2D), and 5-Ethynyl-2'-deoxyuridine (EdU) incorporation assays confirmed the suppression of DNA replication activity (Figure 2E). Moreover, in transwell and wound-healing assays, PUS1 depletion impaired RCC cell migration (Figure 2F, G). To validate these *in vitro* findings, we next assessed the role of PUS1 *in vivo*. Subcutaneous xenograft tumor models showed that PUS1 silencing markedly suppressed tumor growth (Figure 2H-L).

**Figure 3:**
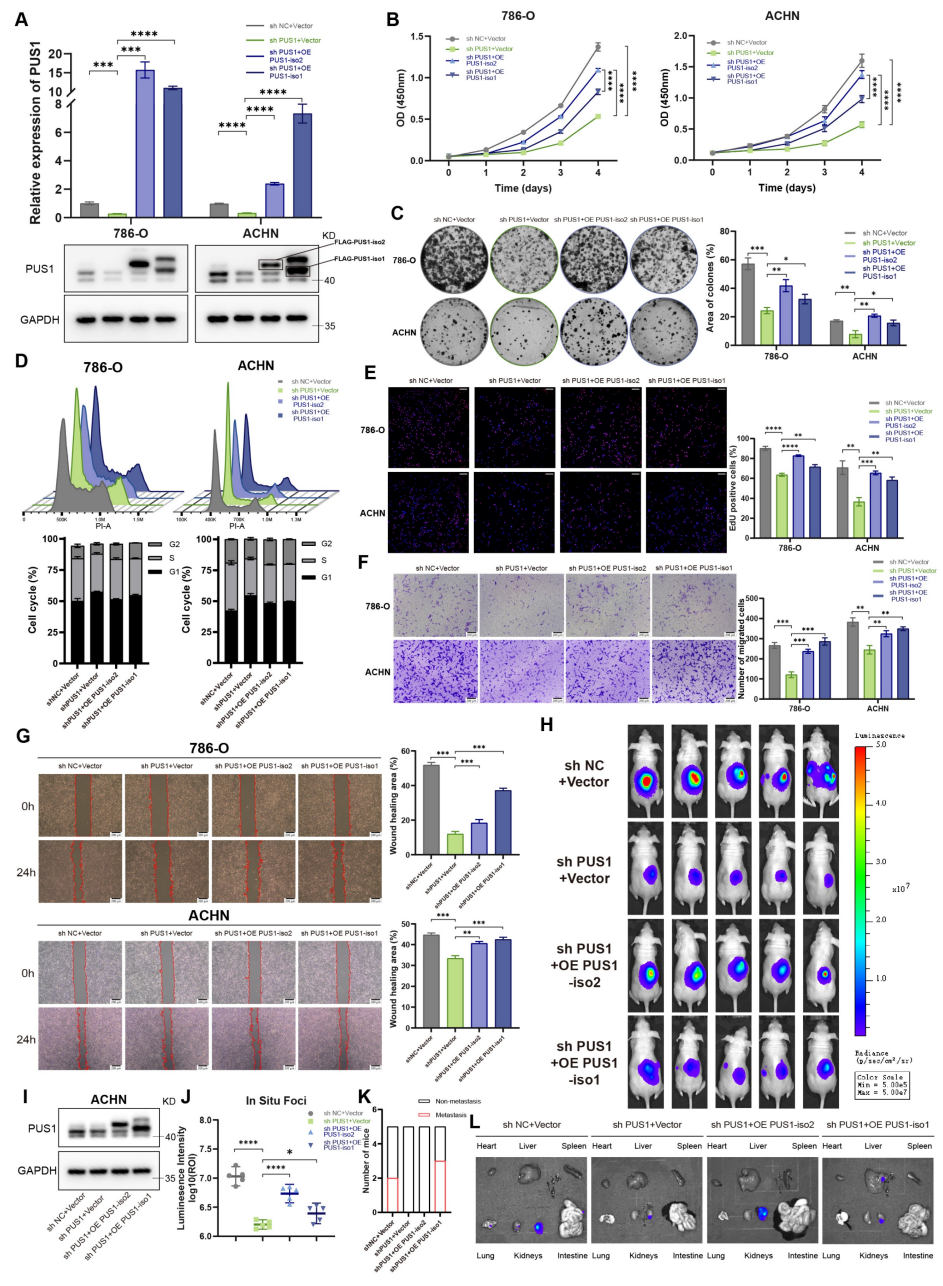
** Re-expression of PUS1 isoform 2 restores proliferative capacity in RCC, whereas PUS1 isoform 1 shows stronger association with metastasis.** (A) RT-qPCR and western blot analysis confirming the re-expression of PUS1 isoforms in 786-O and ACHN cells. (B) Cell proliferation determined by CCK-8 assays following the re-expression of PUS1 isoforms. (C) Representative images of colony-formation assay and its quantification data in 786-O and ACHN cells following PUS1 isoforms re-expression. (D) Flow cytometric analysis of cell cycle and its quantification data in 786-O and ACHN cells following PUS1 isoforms re-expression. (E) Representative images of EdU incorporation assay and its quantification data in 786-O and ACHN cells following PUS1 isoforms re-expression. Scale bar, 200 µm. (F) Representative images of transwell migration assay and its quantification data in 786-O and ACHN cells following PUS1 isoforms re-expression. Scale bar, 200 µm. (G) Representative images of wound healing assay in 786-O and ACHN cells following PUS1 isoforms re-expression. Scale bar, 200 µm. (H) Bioluminescent images showing primary foci and metastasis in mice injected with luciferase-labeled ACHN cells under renal capsule (n = 5). (I) Western blot analysis showing the PUS1 depletion and re-expression in ACHN cells transduced with lentivirus. (J) Bioluminescent signal intensities (photons/s/cm^2^/sr) of primary foci were quantified. (K) Quantification of the number of mice with metastases. (L) Representative bioluminescent images of *ex vivo* organs and metastatic lesions. *P < 0.05, **P < 0.01, ***P < 0.001, ****P < 0.0001; ns, not significant.

Given that the two PUS1 isoforms share substantial sequence identity except at their N-termini, designing isoform-specific siRNAs was not feasible. Therefore, we constructed individual overexpression plasmids for each isoform to study their functions (Figure 3A; Figure S2A). Ectopic expression of either PUS1 isoform 1 or isoform 2 significantly promoted the proliferation and migration of RCC cells (Figure 3B-G; Figure S2B-F). Notably, PUS1 isoform 2 exerted a more pronounced effect on proliferation, whereas isoform 1 had a greater impact on migration. To evaluate their roles in tumor progression *in vivo*, we established orthotopic renal implantation models (Figure 3H, I). In these models, PUS1 knockdown suppressed both primary tumor growth and metastasis. Importantly, the proliferative defect was specifically rescued by the re-expression of PUS1 isoform 2, underscoring its dominant role in driving tumor growth (Figure 3J-L).

Collectively, these data establish PUS1 as a key oncogene in RCC progression, with both isoforms contributing to its oncogenic functions. Given our previous findings that PUS1 isoform 2 is more markedly upregulated in RCC tissues, we focused subsequent investigations primarily on PUS1 isoform 2.

### 3.3 PUS1 promotes RCC cell proliferation in a Ψ-dependent manner

**Figure 4:**
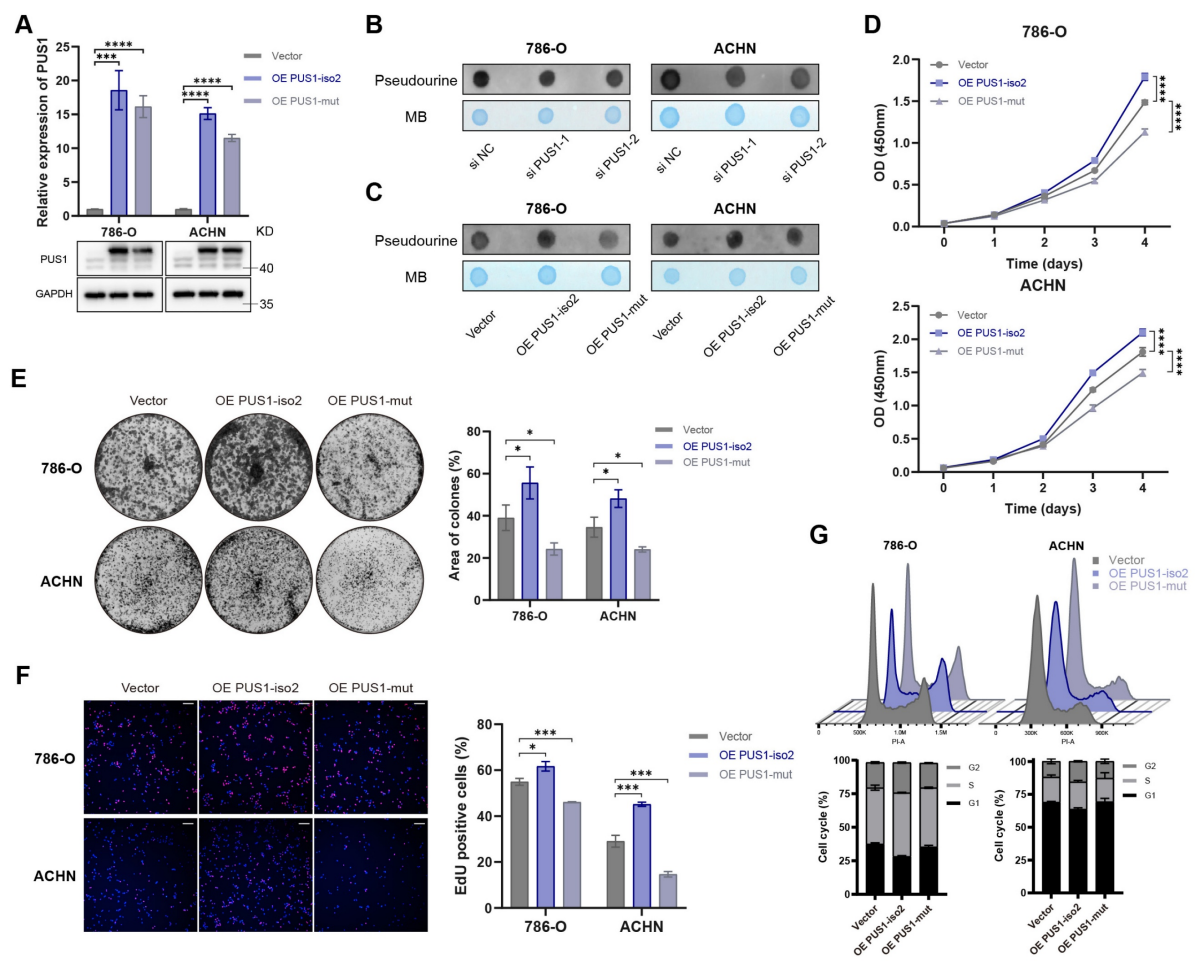
** PUS1 promotes RCC cell proliferation in a pseudouridylation-dependent manner.** (A) RT-qPCR and western blot analysis confirmed the overexpression of PUS1 isoform 2 and its catalytic mutant in 786-O and ACHN cells. (B, C) Dot blot analysis of the Ψ levels of total RNA following PUS1 knockdown (B), or after overexpression of PUS1 isoform 2 and its catalytic mutant(C). Methylene blue (MB) stain served as a loading control. (D) Cell proliferation was determined by CCK-8 assays following the overexpression of PUS1 isoform 2 and its catalytic mutant. (E) Representative images of colony-formation assay and its quantification data in 786-O and ACHN cells following PUS1 isoform 2 or its catalytic mutant overexpression. (F) Representative images of EdU incorporation assay and its quantification data in 786-O and ACHN cells following PUS1 isoform 2 or its catalytic mutant overexpression. Scale bar, 200 µm. (G) Flow cytometric analysis of cell cycle and its quantification data in 786-O and ACHN cells following PUS1 isoform 2 or its catalytic mutant overexpression. *P < 0.05, **P < 0.01, ***P < 0.001, ****P < 0.0001; ns, not significant.

To determine whether the oncogenic function of PUS1 isoform 2 depends on its Ψ modification activity, we constructed a catalytically inactive plasmid with a D118A mutation^25^-^27^ (Figure 4A). Dot blot analysis confirmed that, unlike the wild-type plasmid, the D118A mutant did not alter global Ψ levels (Figure 4B, C). Ectopic expression of wild-type PUS1 significantly promoted the proliferation of RCC cells, whereas the catalytic mutant did not (Figure 4D-G). In fact, ectopic expression of the catalytic mutant even partially suppressed proliferation. These results indicate that the tumor-promoting function of PUS1 isoform 2 is intrinsically dependent on its pseudouridylation activity.

### 3.4 The catalytic activity of PUS1 is essential for global mRNA translation

**Figure 5:**
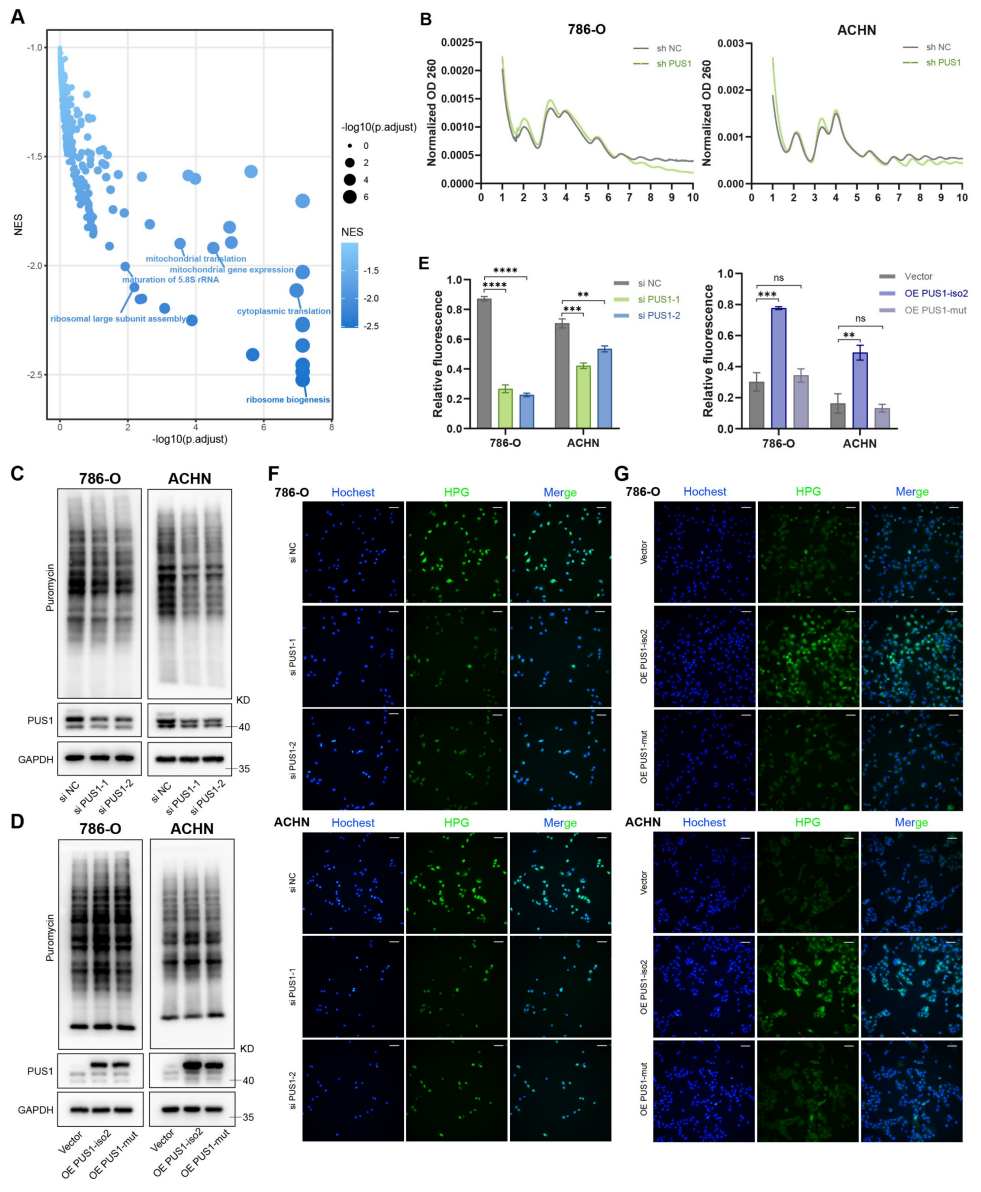
** Depletion of PUS1 inhibits protein synthesis, whereas overexpression of PUS1 isoform 2 enhances it.** (A) Gene set enrichment analysis of downregulated genes in PUS1 knockdown cells. (B) Polysome profiling of control and PUS1 knockdown cells in 786-O and ACHN. (C) Puromycin incorporation assay in 786-O and ACHN cells following PUS1 knockdown. (D) Puromycin incorporation assay in 786-O and ACHN cells following PUS1 isoform 2 or its catalytic mutant overexpression. (E) Quantitative analysis of HPG/Hoechst fluorescence intensity from panels (F) and (G). (F,G) Immunofluorescence images of HPG (green) staining in 786-O and ACHN cells with PUS1 knockdown (F), or with overexpression of PUS1 isoform 2 or its catalytic mutant (G). Nascent protein synthesis was detected by staining with HPG-488 and DNA was stained using Hoechst. Scale bars, 100 µm. *P < 0.05, **P < 0.01, ***P < 0.001, ****P < 0.0001; ns, not significant.

To elucidate the oncogenic mechanism of PUS1, we first performed RNA sequencing in RCC cells following PUS1 knockdown. Gene set enrichment analysis (GSEA) of the downregulated genes revealed significant enrichment of multiple pathways related to protein translation (Figure 5A). Polysome profiling demonstrated a marked reduction in the polysome peak proportion upon PUS1 depletion (Figure 5B), indicating a global inhibition of mRNA translation. This finding was further confirmed by puromycin incorporation assays, which showed a marked reduction in nascent protein synthesis in PUS1-deficient cells (Figure 5C). In contrast, wild-type PUS1 isoform 2, but not the catalytic mutant, markedly increased protein synthesis (Figure 5D). Consistent with these results, a nascent protein synthesis assay provided direct visual confirmation of impaired translation (Figure 5E-G). In summary, these data demonstrate that the catalytic activity of PUS1 is essential for sustaining global protein translation in RCC cells.

### 3.5 PUS1 regulates the translation of downstream proteins independently of its mRNA or tRNA pseudouridylation function

Considering that pseudouridylation can influence translation through modifications of both mRNA and tRNA^28^-^32^, we employed Ribo-seq coupled with quantitative proteomics to identify the primary targets of PUS1. Integrated Ribo-seq and quantitative proteomics identified 40 co-directional genes whose expression was concordantly altered at both the translational and protein level, suggesting translational regulation (Figure S3A; Supplementary Table S1).

We first investigated whether PUS1 directly pseudouridylates the mRNAs of these translationally regulated targets. By referencing published Ψ mapping datasets across multiple groups^14^, ^33^-^35^, we found that only 6 of these 40 genes overlapped with known mRNA pseudouridylation sites (Figure S3B; Supplementary Table S1). Moreover, no overlap was observed between these 40 genes and the known set of PUS1-specific RNA targets (Figure S3C; Supplementary Table S1). Using G6PD as a representative candidate, we confirmed that PUS1 knockdown did not affect its mRNA levels (Figure S3D) but significantly reduced its protein expression (Figure S3E). However, RNA immunoprecipitation (RIP)-qPCR assays with an anti-Ψ antibody demonstrated that PUS1 depletion did not reduce Ψ levels on *G6PD* transcripts in either 786-O or ACHN cells (Figure S3F,G). We further examined Ψ levels on the remaining five candidate genes and observed similar results (Figure S3H-K).

Second, we examined whether PUS1 affects translation through its known role in pseudouridylating a specific set of 26 nuclear tRNAs (Figure S3L)^15^. Given that dysregulation of tRNA modification often alters tRNA abundance or function^36^, we hypothesized that PUS1 knockdown would impair the decoding efficiency of its target tRNAs. We applied the CONCUR pipeline^20^ to examine whether global codon usage differed between PUS1-knockdown and control cells. Increased tRNA abundance and function is expected to shorten ribosomal dwell time at cognate codons^36^. If PUS1 knockdown indeed impaired the functionality or abundance of these tRNAs, ribosomes would stall at codons decoded by PUS1-modified tRNAs, with minimal change at the A+1 site as a control. However, no such stalling signature was detected (Figure S3M-O). Furthermore, comparative analysis of codon usage frequency between transcripts with reduced translation efficiency and others showed no enrichment for codons corresponding to PUS1-targeted tRNAs (Figure S3P-Q). These results collectively indicate that PUS1 regulates the translation of downstream targets through a mechanism distinct from direct mRNA pseudouridylation or modulation of its canonical tRNA substrates.

Collectively, the above results demonstrate that PUS1 promotes global translation independently of direct pseudouridylation of its mRNA or canonical tRNA substrates. This exclusion of the most straightforward mechanisms prompted us to investigate upstream events that could broadly impact translation.

### 3.6 Loss of PUS1 catalytic activity results in the formation of endogenous dsRNA, which in turn inhibits translation

**Figure 6:**
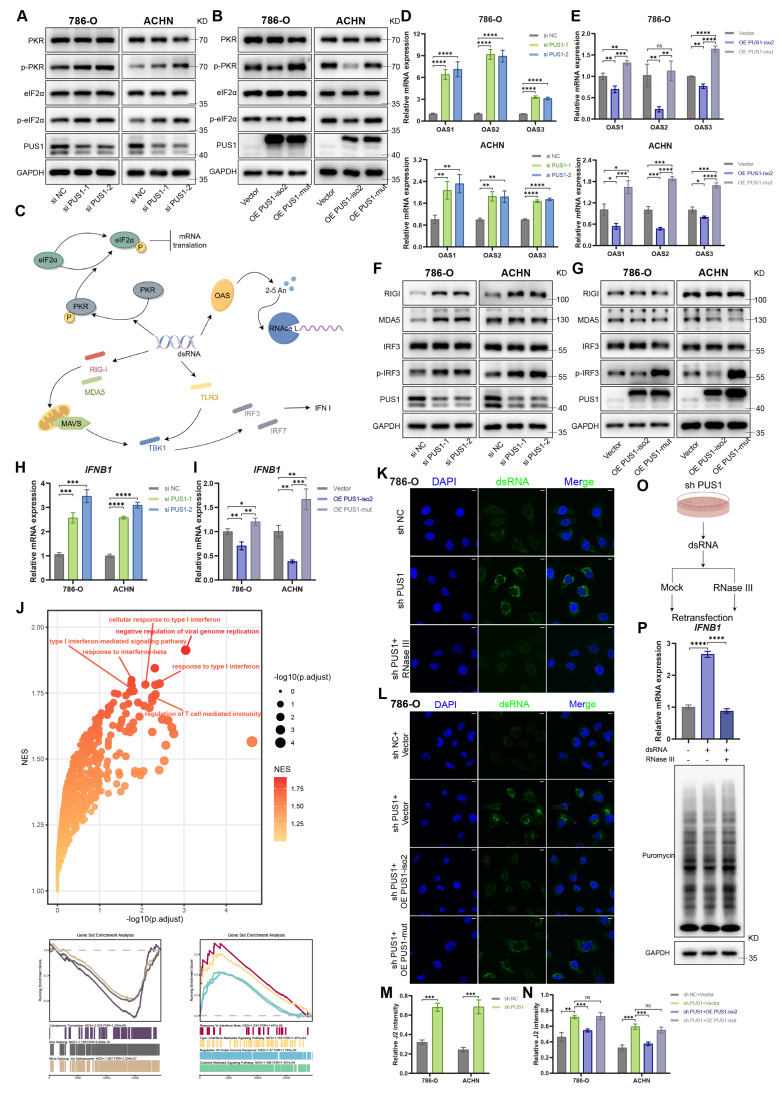
** Depletion of PUS1 results in formation of endogenous dsRNA.** (A,B) Western blotting of p-eIF2α, p-PKR and their corresponding total protein levels in 786-O and ACHN cells following PUS1 knockdown, or after overexpression of PUS1 isoform 2 or catalytic mutant. (C) Schematic diagram of downstream pathways activated by dsRNA. (D,E) RT-qPCR analysis of* OAS1*, *OAS2*, and* OAS3* mRNA expression following PUS1 knockdown, or after overexpression of PUS1 isoform 2 or its catalytic mutant. (F,G) Western blotting of RIGI, MDA5, IRF3, and p-IRF3 protein level in 786-O and ACHN cells following PUS1 knockdown, or after overexpression of PUS1 isoform 2 or its catalytic mutant. (H,I) RT-qPCR analysis of *IFNB1* mRNA expression following PUS1 knockdown, or after overexpression of PUS1 isoform 2 or its catalytic mutant. (J) Gene set enrichment analysis of upregulated genes in PUS1 knockdown cells. (K,L) Immunofluorescence images of dsRNA (green) staining of 786-O cells following PUS1 knockdown (K), or after re-expression of PUS1 isoform 2 or its catalytic mutant (L). dsRNA was detected using J2 antibody, and DNA was stained using DAPI. Scale bars, 10 µm. (M,N) Quantitative analysis of J2 intensity normalized to its DAPI signal. (O) Schematic diagram of dsRNA re-transfection experiment. (P) RT-qPCR analysis of *IFNB1* mRNA expression following re-transfection (top) and puromycin incorporation assay following re-transfection (bottom). *P < 0.05, **P < 0.01, ***P < 0.001, ****P < 0.0001; ns, not significant.

Insights from the role of pseudouridine in mRNA vaccines proved instructive. In that context, Ψ incorporation into 5′-triphosphate RNA potently suppresses protein kinase R (PKR) activation, thereby enhancing translation^37^. This prompted us to examine whether PKR is activated in PUS1-deficient RCC cells. Western blot analysis revealed that PUS1 knockdown robustly induced PKR activation and increased phosphorylation of its substrate, eukaryotic translation initiation factor 2-alpha (eIF2α). In contrast, overexpression of PUS1 isoform 2 but not the mutant suppressed this pathway (Figure 6A, B). PKR can be activated by PACT^38^, or through binding to double-stranded RNA (dsRNA) or 5′-triphosphate RNA^39^-^41^. Given that 5′-triphosphate RNA is minimally generated under physiological conditions, we first investigated whether endogenous dsRNA accumulation triggers PKR pathway activation.

Cellular sensors for dsRNA include RIG-I-like receptors (RLRs), PKR, 2′-5′-oligoadenylate synthetases (OASs), and Toll-like receptors (TLRs), among others (Figure 6C). RT-qPCR analysis revealed upregulated expression of *OASs* in PUS1-knockdown cells (Figure 6D, E). Correspondingly, Western blotting demonstrated elevated protein levels of RIG-I and MDA5, accompanied by increased phosphorylation of interferon regulatory factor 3 (IRF3), a key indicator of type I interferon pathway activation (Figure 6F, G). We further assessed Interferon beta 1 (IFNB1) mRNA expression and found that PUS1 depletion robustly induced *IFNB1* transcription, whereas overexpression of PUS1 isoform 2, but not the catalytic mutant, suppressed its expression (Figure 6H, I). GSEA of upregulated genes from the RNA-seq data further confirmed the enrichment of pathways related to immune and inflammatory responses, as well as cytokine activity, in PUS1-deficient cells (Figure 6J).

To definitively determine if PUS1 deficiency induces dsRNA formation, we stained control and PUS1-deficient renal carcinoma cells using the J2 antibody, which specifically recognizes dsRNA longer than 40 bp^42^. Compared to control, PUS1 knockdown cells exhibited a substantial accumulation of dsRNA (Figure 6K, M; Figure S4A). Overexpression of wild-type PUS1 isoform 2 in PUS1-knockdown cells, but not the catalytic mutant, prevented this dsRNA accumulation (Figure 6L, N; Figure S4B). Next, to assess the immunogenicity of the dsRNA produced upon PUS1 knockdown, we used the J2 antibody to capture dsRNA from shPUS1 cells and transfected this isolated dsRNA into recipient cells (Figure 6O). Crucially, transfection of dsRNA isolated from shPUS1 cells significantly activated *IFNB1* expression and inhibited protein translation in the recipient cells. This effect was completely abolished when the isolated dsRNA was pre-digested with RNase III (which specifically cleaves dsRNA) (Figure 6P). These results provided definitive evidence that dsRNA is primarily responsible for the translational inhibition following the loss of PUS1 catalytic activity.

### 3.7 PUS1 depletion drives the accumulation of dsRNA by promoting intron retention

**Figure 7:**
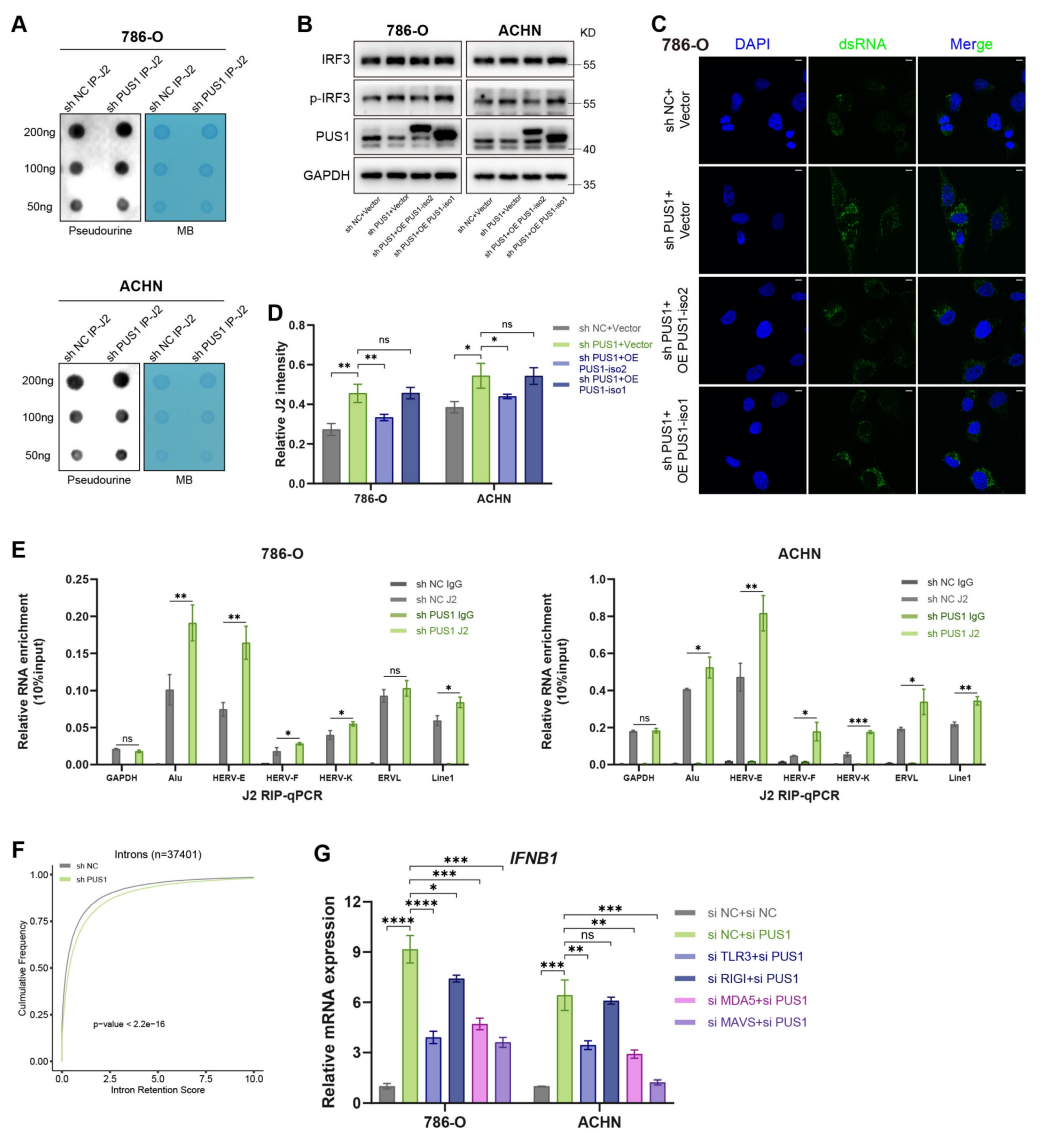
** Origin of dsRNA in PUS1 knockdown cells.** (A) Dot blot analysis of Ψ levels in immunoprecipitated dsRNA from 786-O and ACHN cells following PUS1 knockdown. Methylene blue (MB) staining served as a loading control. (B) Western blot analysis of IRF3 and p-IRF3 protein levels in 786-O and ACHN cells following re-expression of PUS1 isoform 2 or isoform 1. (C) Immunofluorescence images of dsRNA (green) in 786-O cells following re-expression of PUS1 isoform 2 or isoform 1. dsRNA was detected using J2 antibody, and nuclei were stained with DAPI. Scale bars, 10 µm. (D) Quantitative analysis of J2 signal intensity normalized to the DAPI signal. (E) J2-RIP RT-qPCR analysis of the indicated transposable elements. (F) Cumulative distribution curves of the mean intron retention scores in control and PUS1-knockdown groups. (G) RT-qPCR analysis of *IFNB1* mRNA expression in 786-O and ACHN cells transfected with siRNAs targeting Scramble (control), PUS1, or PUS1 in combination with TLR3, RIG-I, MDA5, or MAVS. *P < 0.05, **P < 0.01, ***P < 0.001, ****P < 0.0001; ns, not significant.

Next, we sought to determine the source of dsRNA following PUS1 knockdown. Given that A-to-I and m6A modifications can destabilize dsRNA structures^43^, ^44^, whereas 2′-O-methylation at the 5′ end attenuates the immunogenicity of exogenous transcripts^45^, we first examined whether loss of PUS1 reduces Ψ modification on dsRNA, thereby increasing its abundance or immunogenicity. Using the J2 antibody to isolate dsRNA from shPUS1 and control cells, we performed dot blot analysis to assess Ψ modification levels. The results showed no significant difference in Ψ modification between two groups (Figure 7A).

We then investigated whether dsRNA production was increased. In mammalian cells, dsRNA predominantly originates from mitochondrial RNA and nuclear transposable elements. By overexpressing the two PUS1 isoforms in PUS1-knockdown cells, we found that overexpression of the mitochondrial isoform 1 did not reduce IRF3 phosphorylation (Figure 7B) or dsRNA levels (Figure 7C-D; Figure S5A), whereas the nuclear isoform 2 did. Given their shared catalytic functions but distinct subcellular localizations, we inferred that the dsRNA likely originates primarily from the nucleus. We then used RT-qPCR to measure the level of transcripts from Short Interspersed Nuclear Elements (SINEs), Long Interspersed Elements (LINEs), and Endogenous Retroviruses (ERVs) within the J2-captured dsRNA. The results confirmed significant enrichment in shPUS1 cells compared to controls (Figure 7E). Prior literature indicates that PUS1 can modify pre-mRNA and influence alternative splicing^13^. Consistent with this, our GSEA results indicated significant downregulation of pathways related to mRNA splicing and RNA splicing upon PUS1 knockdown (Figure 6J). Mammalian introns are enriched in transposable elements, particularly SINEs, which constitute major sources of dsRNA^46^, ^47^. Intron retention (IR) analysis revealed a substantial increase in retained introns following PUS1 depletion (Figure 7F). Together, these results demonstrate that dsRNA accumulation in PUS1-deficient cells primarily originates from mis-spliced transcripts. To validate these observations at the transcript level, we analyzed IR events in the *SLC50A1*, *NMRK1*, and *NT5E* transcripts. Agarose gel electrophoresis of RT-PCR products and RT-qPCR revealed elevated IR following PUS1 depletion (Figure S5B-I). The RNA structures predicted by RNAfold (http://rna.tbi.univie.ac.at/cgi-bin/RNAWebSuite/RNAfold.cgi) showed the formation of dsRNA conformation in these retrotransposon-containing introns (Figure S5J-K).

To identify which dsRNA sensor is responsible for detecting PUS1-loss-induced dsRNA and triggering the cellular response, we individually silenced key sensors with siRNAs prior to PUS1 knockdown (Figure S5L). Notably, knockdown of TLR3 or MDA5 significantly decreased the *IFNB1* mRNA expression. Moreover, knockdown of MAVS, the downstream adaptor of the MDA5 pathway, also abolished the type I interferon response induced by PUS1 knockdown (Figure 7G).

### 3.8 PUS1 knockdown synergizes with PD-1 blockade to potently inhibit tumor growth

**Figure 8:**
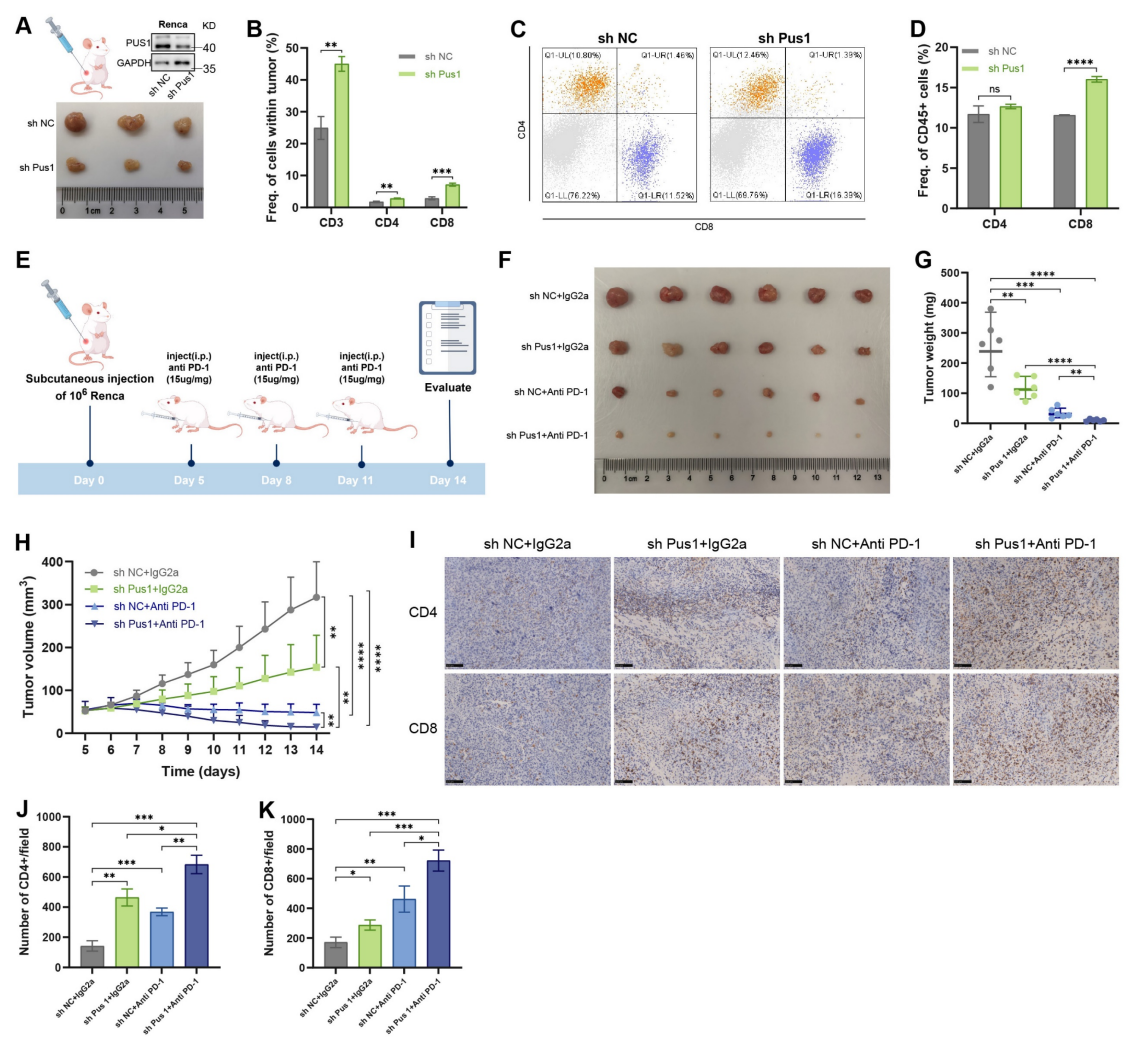
** Pus1 knockdown synergizes with PD-1 blockade.** (A) Tumor tissues for flow cytometry (n = 3). (B) Quantification of CD3^+^, CD4^+^ and CD8^+^ T cells in control and Pus1 depletion Renca tumors. (C,D) Flow cytometric analysis of the proportions of CD4^+^ and CD8^+^ T cell among CD45^+^ cells in control and Pus1 depletion Renca tumors. (E) Schematic diagram of anti-PD-1 treatment timeline. (F-H) Images (F), weights (G), and volumes (H) of xenograft tumors derived from the indicated cells in control, Pus1-knockdown, anti-PD-1, and combination therapy groups (n = 6). (I) Representative IHC staining images for CD4, CD8 in xenograft tumors. Scale bar, 100 µm.(J,K) Quantification of CD4^+^ and CD8^+^ T cells in each treatment group. *P < 0.05, **P < 0.01, ***P < 0.001, ****P < 0.0001; ns, not significant.

Building upon our findings that PUS1 knockdown activates downstream innate antiviral immune responses, we sought to determine whether PUS1 deficiency could consequently enhance tumor susceptibility to anti-tumor immunity. We generated Pus1-deficient Renca cells and implanted them subcutaneously into male BALB/c mice (Figure 8A). Flow cytometric analysis of the tumor immune microenvironment revealed significantly increased infiltration of CD3⁺ T cells, CD4⁺ T cells, and CD8⁺ T cells within Pus1-deficient tumors (Figure 8B,S6A). Moreover, the proportion of CD8⁺ T cells among CD45⁺ cells was also markedly elevated (Figure 8C,D).

Given extensive prior evidence that dsRNA accumulation synergizes with immune checkpoint blockade to enhance antitumor immunity^48^-^50^, we next evaluated the therapeutic potential of combining PUS1 knockdown with PD-1 blockade. Renca cells were implanted subcutaneously into mice, and once tumors formed, mice were treated with either anti-PD-1 antibody or an IgG2a isotype control (Figure 8E). In this model, Pus1 knockdown alone or anti-PD-1 monotherapy each moderately suppressed tumor growth. Strikingly, the combination of Pus1 depletion and PD-1 blockade acted synergistically, markedly delaying tumor progression and even inducing tumor regression in some mice (Figure 8F-H). Furthermore, tumors from the combination therapy group exhibited a significantly higher proportion of tumor-infiltrating CD4⁺ and CD8⁺ T cells (Figure 8I-K).

Collectively, these findings demonstrate that PUS1 depletion enhances antitumor immune responses and strongly sensitizes tumors to PD-1 blockade, highlighting the therapeutic potential of this combination strategy for RCC.

## 4. Discussion

Pseudouridine (Ψ), the earliest discovered post-transcriptional RNA modification, is notable for its abundance, widespread distribution, and high evolutionary conservation^51^, ^52^. Studies on pseudouridine modification have been documented in a range of human cancers, including but not limited to hepatocellular carcinoma, gastric cancer, colorectal cancer, prostate cancer, and glioblastoma^53^. However, its role in renal cell carcinoma (RCC) remains largely elusive. Our analysis of clinical RCC samples revealed a distinct expression pattern for PUS1, highlighting a unique dependency in this cancer type. Unlike malignancies such as prostate cancer and hepatocellular carcinoma, which often exhibit high expression of both PUS1 isoforms^54^, ^55^, RCC exhibits a unique PUS1 dependency pattern, characterized by a low requirement for isoform 1 but a high reliance on the nuclear-localized isoform 2, as evidenced by the marked upregulation of isoform 2 and its critical role in maintaining the proliferative capacity of RCC cells. Nevertheless, isoform 1 demonstrates a robust association with metastasis. Previous evidence has demonstrated that mitochondrial complex I is a key determinant of metastatic potential in renal cancer^56^. The mitochondrial-localized PUS1 targets mitochondrial tRNAs, and its depletion impairs mitochondrial translation, ultimately leading to deficiencies in the function of respiratory chain complexes I and III^57^. We speculate that this may explain why isoform 1 exerts a more pronounced impact on RCC metastasis. However, a technical challenge limited our functional dissection of the individual isoforms. The transcripts of PUS1 isoform 2 and isoform 1 differ only in their 5' untranslated regions, with isoform 1 containing an N-terminal extension that functions as a mitochondrial targeting signal. This high sequence similarity precluded the design of isoform-specific siRNAs. This constraint necessitated the use of overexpression to delineate their individual functions. Additionally, we observed that ectopic expression of the catalytically inactive PUS1 mutant modestly reduced RCC cell proliferation. We speculate that this may reflect a non-enzymatic function of PUS1. This notion is supported by prior work in prostate cancer, where PUS1 was shown to act as a molecular chaperone, protecting downstream effector proteins from ubiquitin-mediated degradation^55^. This potential non-canonical function of PUS1 warrants further investigation.

Post-transcriptional RNA modifications regulate protein synthesis through diverse mechanisms. Research in this field has predominantly focused on mRNA modifications. m6A modification drives malignancy of hepatocellular carcinoma by promoting ATG2A and ATG14 translation^58^. Aberrant m5C hypermethylation confers resistance to gefitinib in EGFR-mutant non-small cell lung cancer by mediating the upregulation of QSOX1 protein synthesis^59^. Our previous work also revealed that m6Am promotes RCC progression by modifying the LPP3 mRNA, thereby enhancing its translation^60^. Consequently, we first investigated the potential role of Ψ modification on mRNA. However, Ψ levels on these target mRNAs remained unchanged following PUS1 perturbation, indicating that their translational regulation occurs through an alternative pathway. Our focus subsequently shifted to tRNA modifications. METTL1-mediated m7G modification of Arg-TCT tRNA can drive oncogenic transformation^61^. In anaplastic thyroid cancer, the m5C methyltransferase NSUN2 can promote codon-dependent oncogenic translation by stabilizing tRNA^62^. PUS1 catalyzes Ψ modifications at positions 27-28 on a specific subset of 26 nuclear tRNAs. A subsequent analysis of our ribosome-seq data revealed no evidence of functional impairment in these tRNAs. We therefore ruled out the possibility that the translation of the identified downstream proteins is influenced by tRNA pseudouridylation. We acknowledge certain limitations to these conclusions. Cellular context can influence RNA modification landscapes and functional outcomes. Specifically, our analysis relied on published Ψ maps from diverse human contexts; the absence of cell-type-specific Ψ-sequencing data for RCC could have obscured relevant modifications. However, this does not compromise the validity of our core conclusions.

The regulation of double-stranded RNA by RNA-modifying enzymes is well-documented. ADAR1 mediates A-to-I editing on cellular dsRNA that can block dsRNA recognition by the MDA5, resulting in subsequent inhibition of autoimmune responses^63^. METTL3-mediated m6A modification destabilizes base-paired RNA duplexes and prevents the formation of endogenous dsRNA and deleterious immune responses during hematopoietic development^64^. NSUN2 depletion leads to a more than threefold increase in RNA abundance of more than 25 genes after dsRNA-IP^65^. Furthermore, NSUN4 induces m5C modification on mitochondrial RNAs (mtRNAs), particularly on the termini of light-strand long noncoding RNAs, thereby promoting RNA turnover and preventing the formation of mitochondrial dsRNA (mt-dsRNA) between the heavy (H-) and light (L-) strands mtRNAs^66^. Our study establishes that PUS1 knockdown likewise triggers dsRNA accumulation, activating the MDA5/RIG-I, PKR-eIF2α, and OAS-RNase L pathways. Through in-depth investigation, we determined that PUS1, unlike ADAR1 or METTL3, does not regulate dsRNA stability through direct modification of the duplexes themselves. This finding is strongly corroborated by an *in vitro* study by Drazkowska, K. *et al.*^67^, which demonstrated that the introduction of Ψ into dsRNA neither altered its thermal stability nor prevented PKR recognition of dsRNA. We next turned our attention to the potential contribution of mt-dsRNA. Mitochondrial DNA is subjected to bidirectional transcription that generates overlapping transcripts (H- and L-strand mtRNAs), which are capable of forming long dsRNA structures. Assessment of the individual isoforms revealed that mitochondrial-localized isoform 1 had a minimal impact on global dsRNA levels, in contrast to isoform 2. Furthermore, in a separate study, Kim, S. *et al.* systematically evaluated various mtRNA-binding proteins, including PUS1, and similarly observed no increase in the expression of H- or L-strand mtRNA^66^. Moreover, our RNA-seq data indicated a broad downregulation of mitochondrial gene expression. This finding provided further supporting evidence for excluding mitochondrial-derived dsRNA as a major contributor. Our investigation then turned to nuclear retrotransposons as the potential dsRNA source. This hypothesis was confirmed by dsRNA-RIP assays, which revealed enriched transposon-derived dsRNA upon PUS1 depletion. Prior studies have demonstrated that PUS1 co-transcriptionally deposits pseudouridines at critical pre-mRNA splicing elements, including splice sites, branch points, and polypyrimidine tracts^13^. Mechanistically, these pseudouridines alter local RNA structural dynamics and reshape the RNA-protein interactome by directly overlapping with the binding motifs of core splicing factors (e.g., U2AF2, SF3A3, and PRPF8). Consequently, the loss of PUS1-mediated modification impairs the proper recruitment of these critical factors, disrupting spliceosome assembly and splice site recognition. This functional impairment perfectly aligns with our observation that PUS1 knockdown led to increased intron retention. This phenomenon mirrors the effect of spliceosome inhibitors, which are known to produce immunogenic dsRNA^46^. Collectively, these findings allowed us to establish the mis-spliced mRNA as the primary source of dsRNA accumulating upon PUS1 loss.

Finally, to unequivocally define the therapeutic relevance of PUS1 in tumors, we turned to an immunocompetent mouse model. Subcutaneous implantation of Pus1-deficient tumors demonstrated that Pus1 loss robustly enhances the infiltration of both CD4⁺ and CD8⁺ T cells. Furthermore, Pus1 depletion synergized with PD-1 blockade to potentiate tumor cell killing. This discovery opens new avenues for therapeutic intervention in renal cell carcinoma, paving the way for novel treatment modalities.

## 5. Conclusion

In summary, our study identifies PUS1 as a crucial epigenetic regulator that promotes renal cell carcinoma progression. Mechanistically, the presence of PUS1 limits dsRNA levels, inhibits the activation of the innate antiviral immune system, then prevents cellular translational arrest, and reduces immune system activation.

## Supplementary Material

Supplementary figures.

Supplementary table 1: Venn diagram.

Supplementary table 2: clinical characteristics.

Supplementary table 3: siRNAs and shRNAs.

Supplementary table 4: primers.

Supplementary table 5: antibodies.

## Data Availability

The RNA-seq data between PUS1 knockdown vs control in RCC cells have been deposited in the Genome Sequence Archive in National Genomics Data Center, China National Center for Bioinformation / Beijing Institute of Genomics, Chinese Academy of Sciences (GSA-Human: HRA0014674) that are available upon reasonable request.

## Abbreviations

Ψ: Pseudouridine
PUS1: Pseudouridine synthase 1
RCC: Renal cell carcinoma
dsRNA: double-stranded RNA
mt-dsRNA: mitochondrial dsRNA
TKI: Tyrosine kinase inhibitors
ICIs: Immune checkpoint inhibitors
OS: Overall survival
FBS: Fetal bovine serum
CCK-8: Cell Counting Kit-8
RIP: RNA Immunoprecipitation
IHC: Immunohistochemistry
IR: Intron retention
GSEA: Gene set enrichment analysis
PKR: Protein kinase R
eIF2α: the substrate eukaryotic translation initiation factor 2-alph
RLRs: RIG-I-like receptors
OASs: 2′-5′-oligoadenylate synthetases
TLRs: Toll-like receptors
IRF3: Interferon regulatory factor 3
IFNB1: Interferon beta 1
SINEs: Short Interspersed Nuclear Elements
LINEs: Long Interspersed Elements
ERVs: Endogenous Retroviruses
