# Supplementary material for: PUS1 Drives Renal Cancer Progression by Preventing Formation of Endogenous Double-stranded RNAs
Source: Int J Biol Sci. 2026 Mar 25;22(7):3749–68. doi: 10.7150/ijbs.130175 (PMC13086011; doi:10.7150/ijbs.130175)
Supplement: Supplementary file 1 — Supplementary figures. [file ijbsv22p3749s1.pdf]

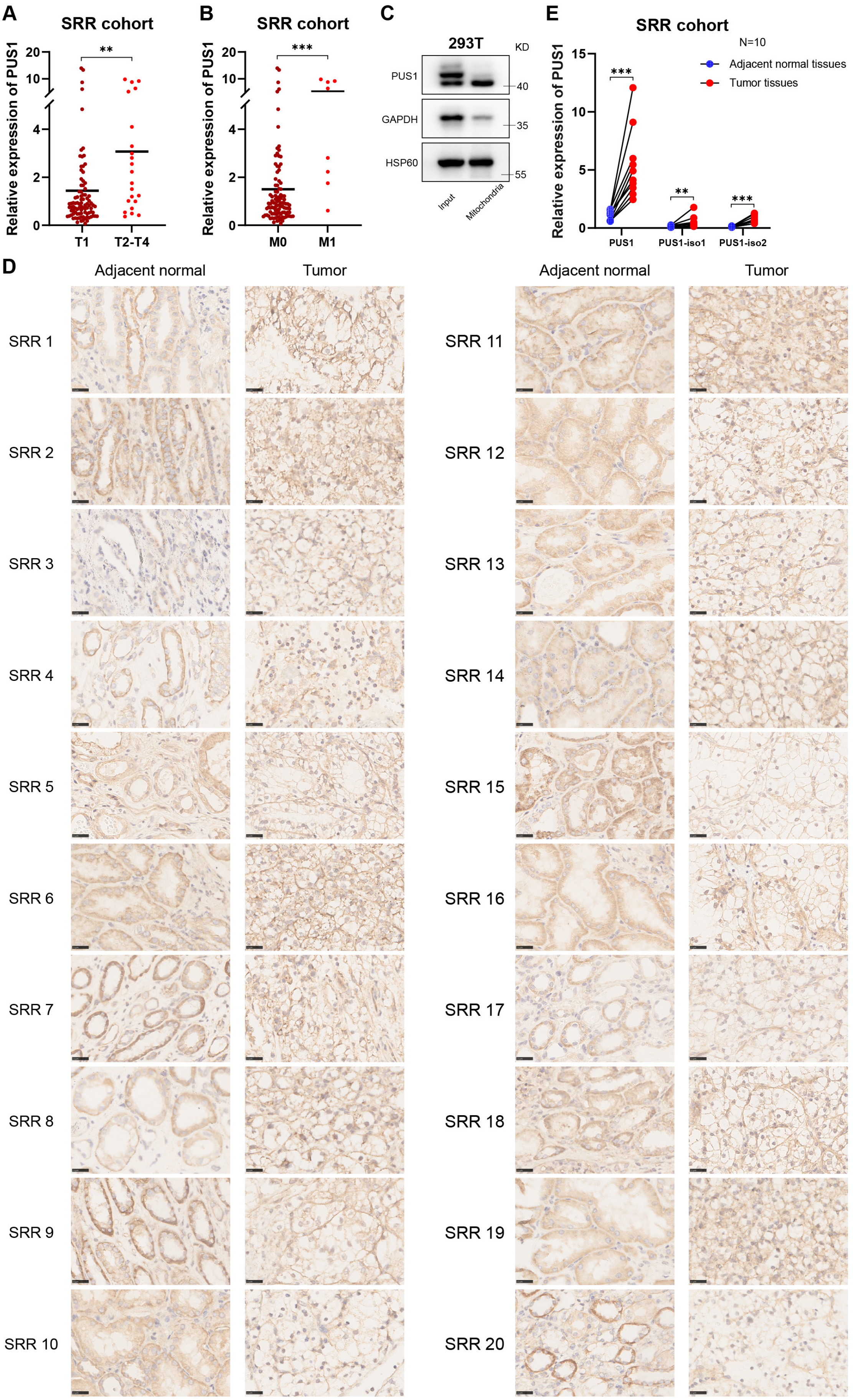
A
SRR cohort
Relative expression of PUS1
T1
T2-T4
**
B
SRR cohort
M0
M1
***
C
293T
KD
PUS1
GAPDH
HSP60
40
35
55
Input
Mitochondria
E
SRR cohort
N=10
Adjacent normal tissues
Tumor tissues
PUS1
PUS1-iso1
PUS1-iso2
D
Adjacent normal
Tumor
SRR 1
SRR 2
SRR 3
SRR 4
SRR 5
SRR 6
SRR 7
SRR 8
SRR 9
SRR 10
SRR 11
SRR 12
SRR 13
SRR 14
SRR 15
SRR 16
SRR 17
SRR 18
SRR 19
SRR 20

**Figure S1. PUS1 expression level from the internal RCC cohort.** (A) *PUS1* expression level between T1 stage and T2-T4 stage from the SRR RCC cohort. (B) *PUS1* expression level between non-metastasis and metastasis groups from the SRR RCC cohort. (C) Western blot analysis of PUS1 and different markers in the mitochondrial fractions of 293T cells. (D) Part of IHC staining images for PUS1 protein in the SRR RCC cohort are presented. (E) RT-qPCR analysis of *PUS1*, *PUS1*-iso1, and *PUS1*-iso2 in RCC specimens from the SRR RCC cohort. *P < 0.05, **P < 0.01, ***P < 0.001, ****P < 0.0001; ns, not significant.

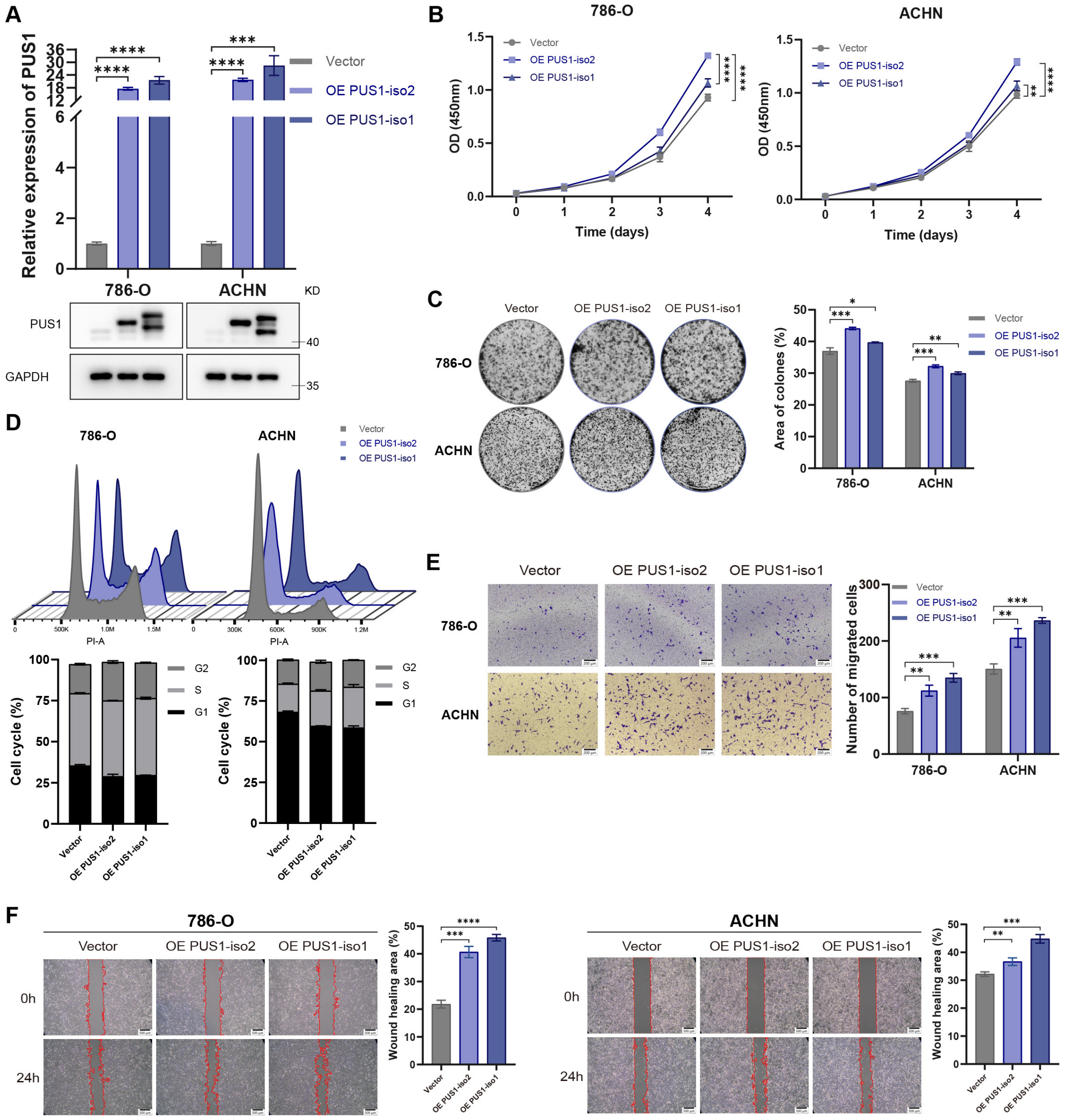


**Figure S2. Overexpression of PUS1 isoform 2 exerts a more pronounced effect on proliferation.** (A) RT-qPCR and western blot analysis confirming the overexpression of PUS1 isoforms in 786-O and ACHN cells. (B) Cell proliferation determined by CCK-8 assays following the overexpression of PUS1 isoforms. (C) Representative images of colony-formation assay and its quantification data in 786-O and ACHN cells following PUS1 isoforms overexpression. (D) Flow cytometric analysis of cell cycle and its quantification data in 786-O and ACHN cells following PUS1 isoforms overexpression. (E) Representative images of transwell migration assay and its quantification data

in 786-O and ACHN cells following PUS1 isoforms overexpression. Scale bar, 200 μm. (F) Representative images of wound healing assay in 786-O and ACHN cells following PUS1 isoforms overexpression. Scale bar, 200 μm. *P < 0.05, **P < 0.01, ***P < 0.001, ****P < 0.0001; ns, not significant.

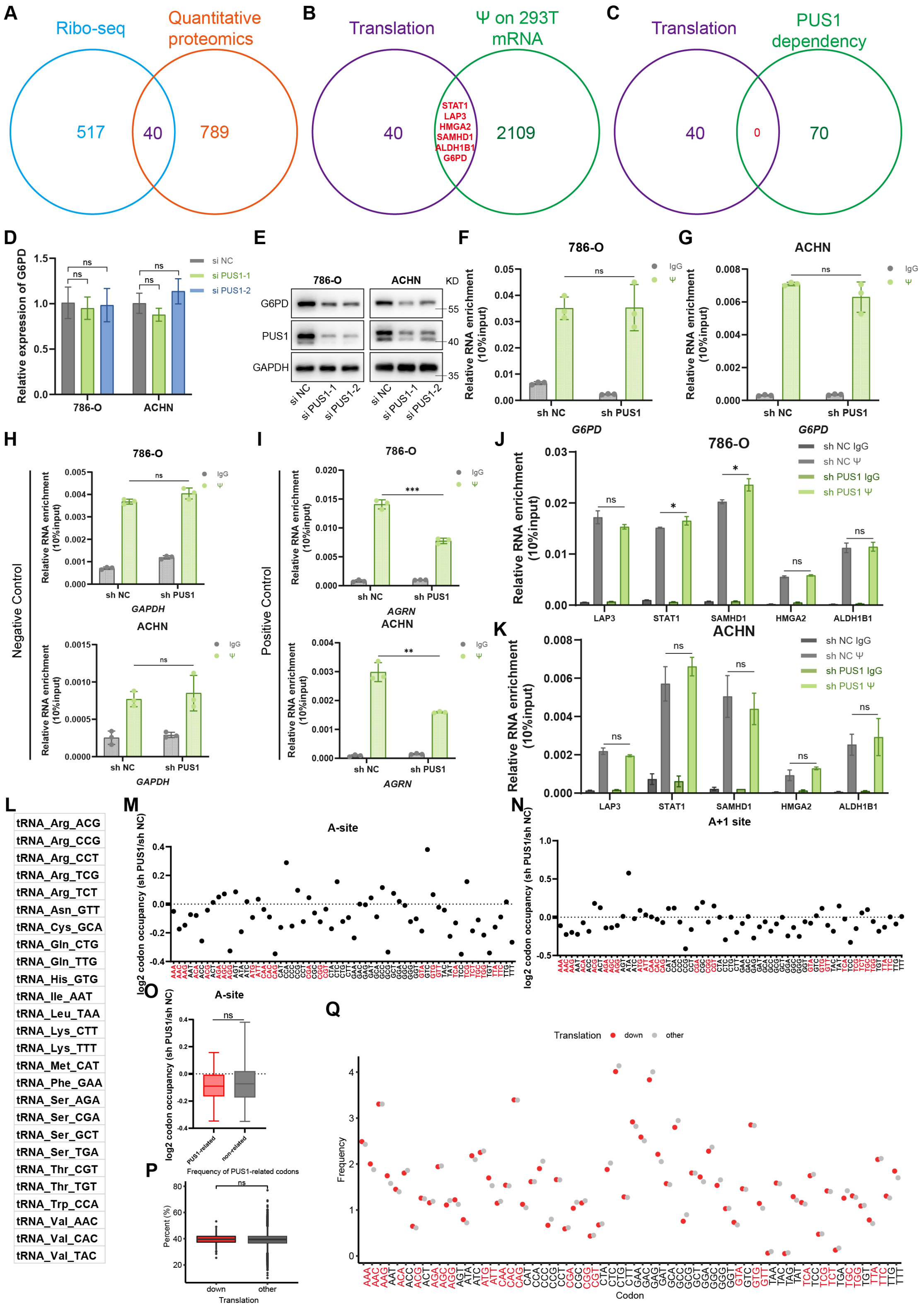


**Figure S3. PUS1-mediated translation of specific targets is independent of its**

**pseudouridylation roles in mRNA or tRNA.** (A) Venn diagram showing the overlap of differentially expressed genes from Ribo-seq and quantitative proteomics results. (B, C) Venn diagram showing the overlap among Ribo-seq, quantitative proteomics, and Ψ-sequencing results. (D) RT-qPCR analysis of *G6PD* mRNA levels in 786-O and ACHN cells transfected with the indicated PUS1 siRNAs. (E) Western blot analysis of G6PD protein levels in 786-O and ACHN cells transfected with the indicated PUS1 siRNAs. (F, G) Ψ-RIP assay showing the Ψ levels on *G6PD* in 786-O and ACHN cells following PUS1 knockdown. (H, I) Positive control and negative control of Ψ-RIP assay.(J, K) Ψ-RIP assay showing the Ψ levels on candidate genes as indicated in 786-O and ACHN cells following PUS1 knockdown. (L) List of tRNAs modified by PUS1. (M, N) Ribosome occupancy at individual codons at A sites and A+1 sites. The codons are separated into PUS1-related(red) and not PUS1-related (black) groups. (O) Analysis of ribosome occupancy at individual codons in A site from PUS1-related tRNA and non-PUS1-related tRNA.(P,Q) Frequency of PUS1-related tRNA-decoded codons in genes with decreased translation efficiency (down), and other genes (other) in PUS1-depleted cells. $^{*}P < 0.05$, $^{**}P < 0.01$, $^{***}P < 0.001$, $^{****}P < 0.0001$; ns, not significant.

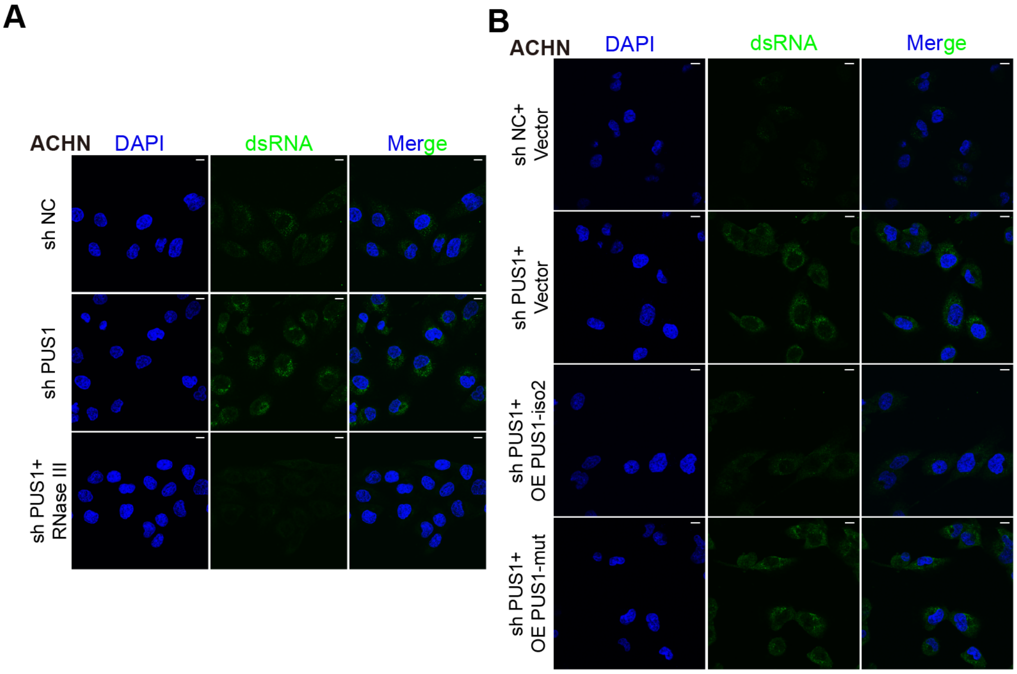


**Figure S4. Immunofluorescence images** (A,B) Immunofluorescence images of dsRNA (green) staining of ACHN cells following PUS1 knockdown (A), or after re-expression of PUS1 isoform 2 or its catalytic mutant (B). dsRNA was detected using J2 antibody, and DNA was stained using DAPI. Scale bars, 10 μm.

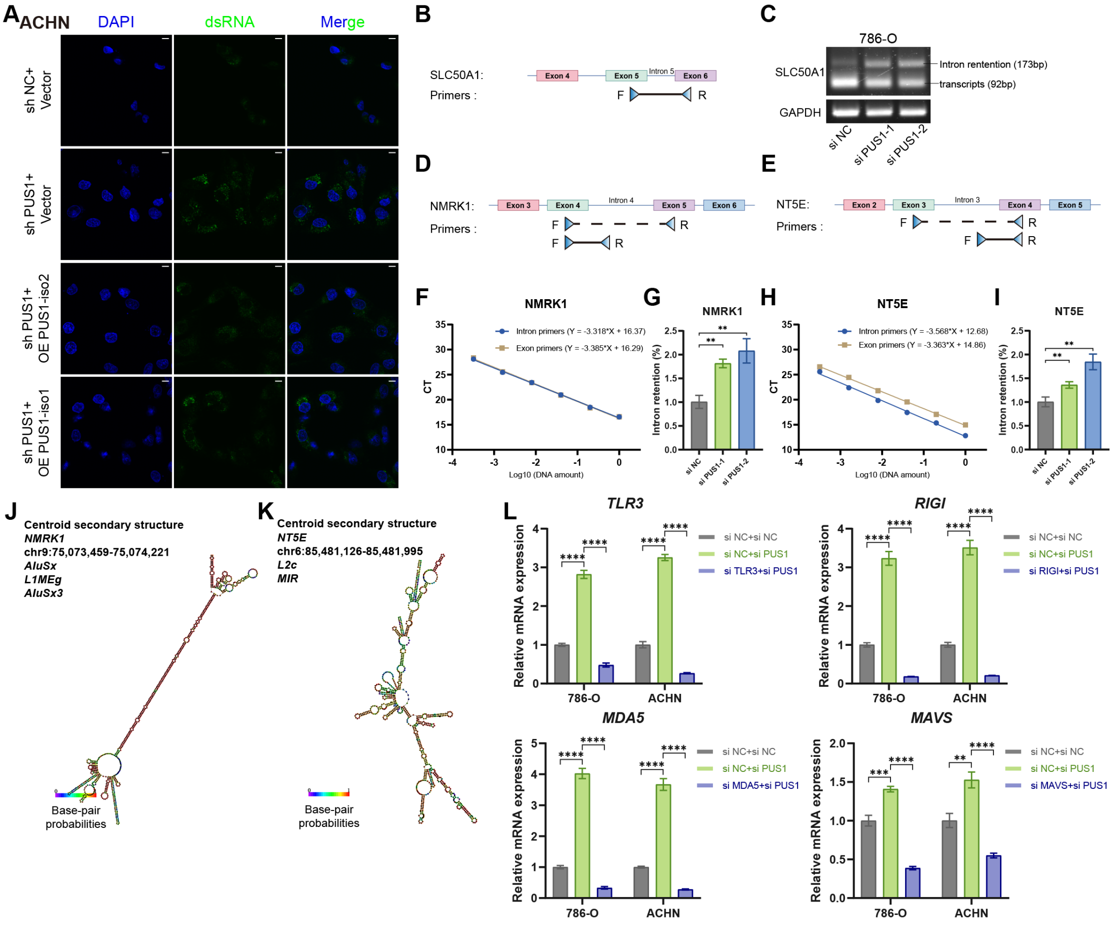

**Figure S5 Origin of dsRNA.** (A) Immunofluorescence images of dsRNA (green) in ACHN cells following re-expression of PUS1 isoform 2 or isoform 1. dsRNA was detected using J2 antibody, and nuclei were stained with DAPI. Scale bars, 10 μm. (B) The schematic of the patterns of intron retention and the designed primers of *SLC50A1*. (C) Intron retention of *SLC50A1* in 786-O cells following PUS1 knockdown measured by agarose gel electrophoresis. (D,E) The schematic of the patterns of intron retention and the designed primers of *NMRK1* and *NT5E*. Detection primers—intron-retaining transcripts, and reference primers—total transcripts of the gene. (F,H) Primer efficiencies determined by qPCR of purified primer amplicons at serial dilutions. Calculated primer efficiencies using formula E = -1+10(-1/slope) are indicated. (G,I) Intron retention of *NMRK1* and *NTE5* in 786-O cells following PUS1 knockdown measured by RT-qPCR. (J,K) Retrotransposon-containing introns in *NMRK1* and *NTE5* are predicted to form double-stranded secondary structures by RNAfold. (L) RT-qPCR analysis confirming the knockdown of TLR3, RIGI, MDA5, or MAVS in 786-O and ACHN cells.

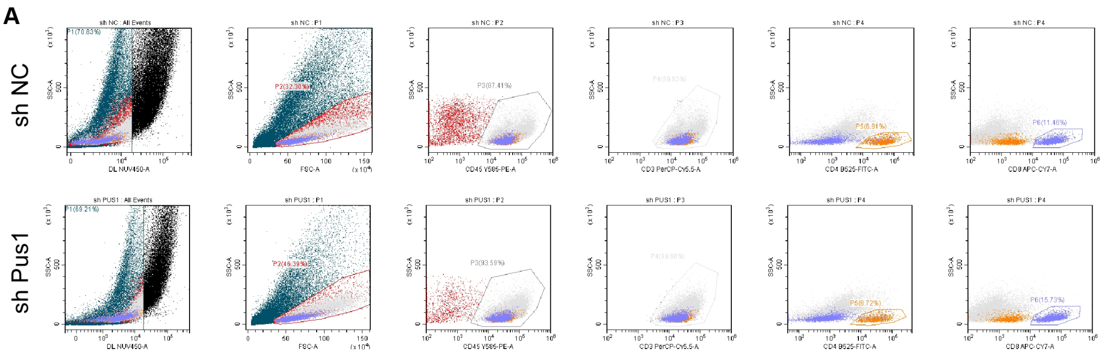


**Figure S6. Flow cytometry gating strategies and representative plots.** (A) Gating strategy and representative flow cytometry plots for the assessment of $CD3^+$, $CD4^+$, and $CD8^+$ T cells in control and Pus1 depletion Renca tumors.
